# A Companion Cell–Dominant and Developmentally Regulated H3K4 Demethylase Controls Flowering Time in *Arabidopsis* via the Repression of *FLC* Expression

**DOI:** 10.1371/journal.pgen.1002664

**Published:** 2012-04-19

**Authors:** Hongchun Yang, Zhifu Han, Ying Cao, Di Fan, Hong Li, Huixian Mo, Yi Feng, Lei Liu, Zheng Wang, Yanling Yue, Sujuan Cui, She Chen, Jijie Chai, Ligeng Ma

**Affiliations:** 1 State Key Laboratory of Plant Physiology and Biochemistry, College of Biological Sciences, China Agricultural University, Beijing, China; 2 National Institute of Biological Sciences, Beijing, China; 3 Hebei Key Laboratory of Molecular Cell Biology, College of Biological Sciences, Hebei Normal University, Shijiazhuang, China; 4 College of Life Sciences, Capital Normal University, Beijing, China; Gregor Mendel Institute of Molecular Plant Biology GmbH, Austria

## Abstract

Flowering time relies on the integration of intrinsic developmental cues and environmental signals. FLC and its downstream target *FT* are key players in the floral transition in *Arabidopsis*. Here, we characterized the expression pattern and function of *JMJ18*, a novel JmjC domain-containing histone H3K4 demethylase gene in *Arabidopsis*. *JMJ18* was dominantly expressed in companion cells; its temporal expression pattern was negatively and positively correlated with that of *FLC* and *FT*, respectively, during vegetative development. Mutations in *JMJ18* resulted in a weak late-flowering phenotype, while *JMJ18* overexpressors exhibited an obvious early-flowering phenotype. JMJ18 displayed demethylase activity toward H3K4me3 and H3K4me2, and bound *FLC* chromatin directly. The levels of H3K4me3 and H3K4me2 in chromatins of *FLC* clade genes and the expression of *FLC* clade genes were reduced, whereas *FT* expression was induced and the protein expression of FT increased in *JMJ18* overexpressor lines. The early-flowering phenotype caused by the overexpression of JMJ18 was mainly dependent on the functional FT. Our findings suggest that the companion cell–dominant and developmentally regulated JMJ18 binds directly to the *FLC* locus, reducing the level of H3K4 methylation in *FLC* chromatin and repressing the expression of *FLC*, thereby promoting the expression of *FT* in companion cells to stimulate flowering.

## Author Summary

Flowering is an important developmental transition during plant life cycle and the key process for production of the next generation. Flowering time is controlled by both intrinsic developmental and environmental signals. FLC and its target *FT* work as repressor and activator, respectively, to regulate flowering time in *Arabidopsis*; thus the regulation of *FLC* and *FT* expression is the key for the control of floral transition. Epigenetic modifications are critical for transcription regulation. Here, we show that a novel JmjC domain-containing histone H3K4 demethylase, JMJ18, is a key regulator for the expression of *FLC* and *FT* in companion cells and flowering time. *JMJ18* is dominantly expressed in vascular tissue; its temporal expression pattern was developmentally regulated, and negatively and positively correlated with *FLC* and *FT*, respectively. *JMJ18* mutation leads to weak late-flowering, while *JMJ18* overexpressor exhibited an obvious early-flowering phenotype. JMJ18 binds to chromatin of *FLC*, represses its expression, and releases expression of *FT* in companion cells. Our results suggest that JMJ18 is a developmentally regulated companion cell–dominantly expressed signal to control flowering time by binding to *FLC*—reducing level of H3K4 methylation in *FLC* and repressing expression of *FLC*—thereby promoting expression of *FT* in companion cells during vegetative development in *Arabidopsis*.

## Introduction

DNA is packaged as chromatin in eukaryotic cells. Nucleosomes, which consist of an octamer of four histones wrapped around 146 base pairs of DNA, are the fundamental unit of chromatin [1]. The flexible N-terminal tails of histones, which protrude from the nucleosome core particle, are subject to several types of covalent modification, including acetylation, methylation, phosphorylation, and ubiquitylation [2]. Each of these modifications is reversible and is required for the dynamic regulation of gene expression [3].

*In vivo*, the lysine residues in histones display three distinct methylation states: mono- (me1), di- (me2), and tri-methylated (me3). These differences in methylation are important for the recognition of chromatin by chromatin modulators and for the recruitment of other modulators and regulators [4], 5,6,7,8. In addition, lysine methylation at different sites within the histone protein plays distinct roles in gene activation and repression [2], [9]. For example, the methylation of H3K4 and H3K36 is correlated with gene activation, while transcriptional repression has been demonstrated in genomic regions with increased levels of H3K9 and H3K27 methylation [3], [10], [11], [12]. Various developmental processes are regulated by histone methylation in animals and plants. For example, H3K27 methylation mediated by the PRC2 complex is required for embryonic development and stem cell identity in mammals and controls most steps in the development of *Arabidopsis* [9], [13], [14], [15].

The methylation state of histone proteins is determined by the balance between methylation and demethylation, which is mediated by histone methyltransferases and demethylases, respectively [16], [17], [18]. However, the reversibility of histone methylation *in vivo* is the latest to be discovered compared to other covalent forms of histone modification. The amine oxidase LSD1 was the first histone demethylase found to demethylate H3K4me2 and H3K4me1 through an FAD-dependent oxidation reaction [18]. LSD1 demethylase family proteins are unable to remove methyl groups from tri-methylated lysines, suggesting the presence of other histone demethylases in eukaryotic cells [18]. More recently, a family of JmjC domain-containing proteins was characterized as histone demethylases which were able to reduce any one of the three histone lysine methylation states at several specific sites in yeast and animals [17], [19], [20], [21]. These histone demethylases are involved in many biological processes in animals, including spermatogenesis, *HOX* gene regulation, and germ cell development [19], [22], [23].

FLD and LDLs are the homologs of human LSD1 in *Arabidopsis*. They promote the floral transition by constitutively inhibiting the expression of *FLC* [24], [25], [26]. The *Arabidopsis* genome contains 21 JmjC family proteins [27]; however, only five of them have been characterized, and they have been found to be involved in RNA silencing, DNA methylation, flowering time control, circadian clock regulation, BR signaling and shoot regeneration *in vitro* [28], [29], [30], [31], [32], [33], [34], [35], [36].

Flowering at the appropriate time is the most important factor in achieving reproductive success. To produce the next generation, plants rely on intricate signaling pathways involving a variety of intrinsic factors, including developmental stage and age, and environmental cues such as photoperiod, temperature, and light quantity [37], [38], [39]. Thus, distinct environmental conditions modulate endogenous gene expression to ensure flowering at the correct time [12], [40].

*FLOWERING LOCUS C* (*FLC*) encodes a MADS-box transcription factor and floral repressor that regulates flowering time in a dosage-dependent manner by integrating the vernalization, autonomous, PAF1 complex, and H2B ubiquitination pathways [11], [41], [42], [43], [44], [45]. *FLC* and the functional locus *FRIGIDA* (*FRI*) act together to produce winter-annual *Arabidopsis* accessions, which must be exposed to cold temperature for several weeks to repress *FLC* expression and promote flowering in the following spring [46], [47]. As endogenous factors, autonomous pathway genes consecutively repress *FLC* expression [48], [49]. In addition, the *FLC* antisense transcript affects the expression of sense transcript, thereby influencing flowering time in *Arabidopsis* [50], [51], [52].

*FLC* expression is predominant in the shoot apical meristem (SAM); however, it is also expressed in vascular tissue in young leaves and the root tip [44], [53], [54]. *FLC* expressed in the SAM and leaf vascular tissue contributes to the control of flowering time in *Arabidopsis* [55]. During vernalization, VIN3 binds *FLC* chromatin, thereby repressing its expression in the SAM [53]. The components of the autonomous pathway repress, while those of the PAF1 complex activate, *FLC* expression in the SAM [11], [37], [43]. Thus, although the regulation of *FLC* expression in the SAM has been extensively studied, little is known about how *FLC* expression is regulated in leaf vascular tissue.

Histone modification plays crucial roles in the regulation of *FLC* expression in *Arabidopsis*. In *FLC* chromatin, H3K4 hyper-tri-methylation and acetylation are associated with the activation of gene expression [43], [56], [57], [58]. The methylation of H3K27 and H3K9 in *FLC* chromatin leads to the repression of *FLC* expression and is required for maintenance of the repression of *FLC* expression in plants growing in the following spring after vernalization [12], [53], [59]. In addition, H2B monoubiquitination is required for the maintenance of high levels of H3K4me3 and H3K36me2 [11], [45]. A loss of H2B monoubiquitination decreases the level of H3K4me3 and H3K36me2, leading to the repression of *FLC* transcription and early flowering [11], [45].

FLOWERING LOCUS T (FT), which is a component of the photoperiod pathway, coordinates signals from the vernalization, autonomous, PAF1 complex, and photoperiod pathways to promote flowering in response to increase in day length [60], [61], [62], [63]. *FT* expression is restricted to leaf phloem companion cells [62]. FT travels from leaves to the SAM, where it interacts with the bZIP transcription factor FD to stimulate floral meristem initiation [64], [65], [66], [67], [68]. As a mobile and systemic signal, FT integrates photoperiod- and *FLC*-dependent pathways to control flowering time by regulating the expression of floral identity genes. It was shown previously that the companion cells specifically expressed FLC represses *FT* expression in leaf companion cells and delays the expression of its cofactor *FD* in the SAM [55]. Thus, FLC systemically blocks the function of FT to repress flowering. However, the regulation of *FLC* expression in companion cells for the control of floral development has not been characterized.

As mentioned above, several JmjC domain-containing proteins have been reported to be involved in flowering time control [28], [29], . Among them, JMJ14 is a member of JARID family with H3K4 demethylase activity, and is involved in flowering time control through the repression of floral integrators [28], [29], [33], [69]. It is not yet clear whether those floral integrator genes are the direct targets of JMJ14 [28], [29], [69]. ELF6 acts as a repressor in photoperiod pathway [31], and its closest homolog REF6 acts as a *FLC* repressor in flowering time regulation [31]. Recent results suggest that REF6 is a H3K27 demethylase; thus, *FLC* is not likely to be the direct target of REF6 [70]. No JmjC domain-containing histone demethylases that target *FLC* locus have yet been found.

In this paper, we describe the expression pattern and function of JMJ18, a novel JmjC domain-containing histone H3K4 demethylase in *Arabidopsis*. *JMJ18* was predominant expressed in phloem companion cells, and its level of expression increased during vegetative development. JMJ18 was found to promote the floral transition in *Arabidopsis* by binding *FLC* chromatin and demethylating H3K4 methylation, leading to the repression of *FLC* and enhanced expression of the downstream flowering activator *FT* in companion cells.

## Results

### JMJ18 demethylates histone H3K4me3 and H3K4me2 *in vitro*

JMJ18 (At1g30810) belongs to the evolutionarily conserved JARID1 family. Previous studies have shown that JARID1 family proteins demethylate H3K4 methylations in yeast, animal and *Arabidosis* [28], [29], [33], [69], [71], [72], [73].

**Figure 1:**
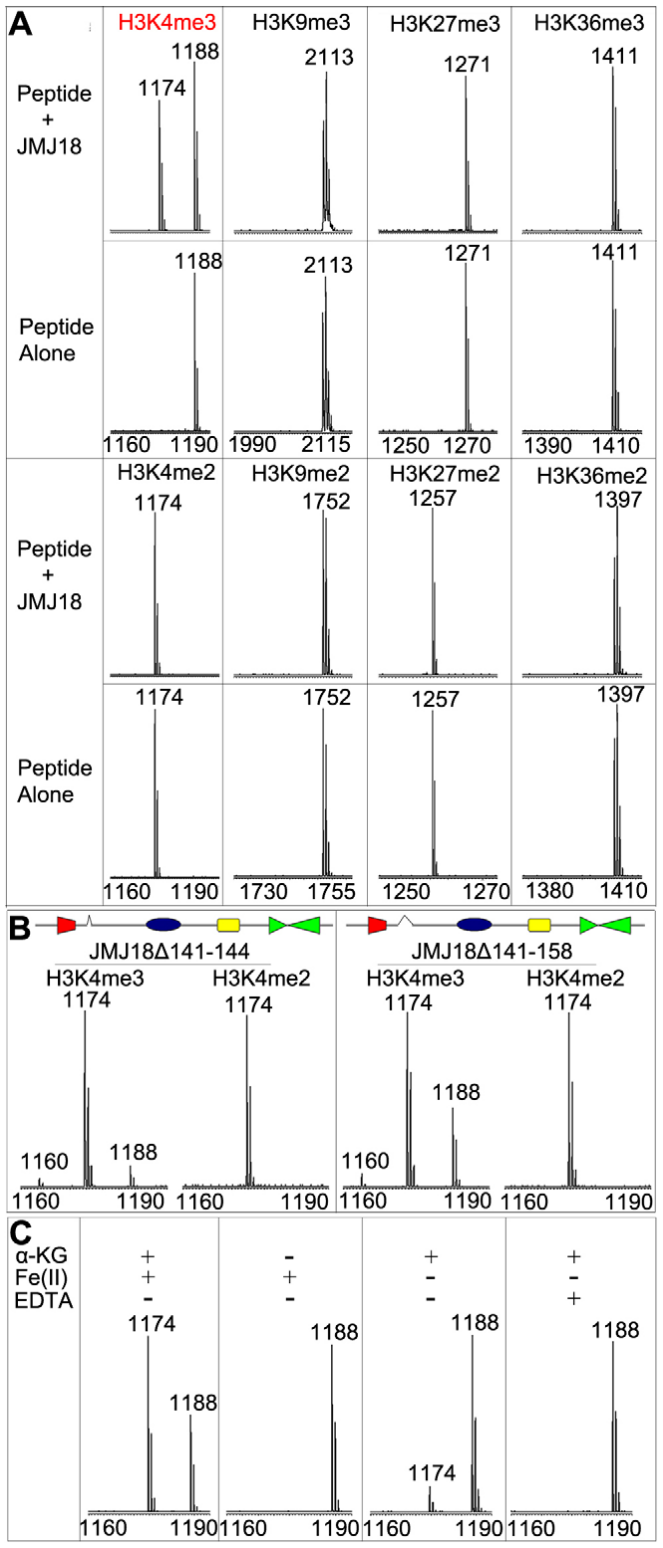
Mass spectrometric analysis of JMJ18 histone demethylase activity *in vitro*. (A) Characterization of JMJ18 histone demethylase site specificity using purified full-length JMJ18. The numbers represent the molecular weights of the peptides. (B) Histone demethylase activity of two truncated forms of JMJ18: His-JMJ18Δ141–144 and His-JMJ18Δ141–158. Tri- and di-methylated H3K4 peptides were used in the assay. Both truncated JMJ18 were able to demethylate tri-methylated H3K4 to the di- and mono-methylated states; however, no demethylase activity was detected if di-methylated H3K4 peptide was used as the substrate. The deleted domain was indicated by an open arrow in the schematic structure of JMJ18. (C) The histone demethylase activity of JMJ18 depends on α-KG and Fe(II).

To determine whether JMJ18 exhibits histone demethylase activity, we expressed recombinant His-tagged JMJ18 and purified the recombinant protein from insect cells (Figure S1A and S1B). A MALDI-TOF mass spectrometric analysis-based demethylation assay was used to detect the histone demethylation activity of JMJ18. Purified His-JMJ18 was incubated with a variety of histone peptides representing the tri- and di-methylated states of the four lysine residues in histone H3. As shown in Figure 1A, JMJ18 converted H3K4me3 to H3K4me2, but did not alter the H3K4me2 methylation state or the methylation status of any other lysine residue in the peptides (summarized in Figure S1C).

To investigate the demethylase activity of JMJ18 further, we purified two truncated versions of JMJ18, JMJ18Δ141–144 and JMJ18Δ141–158, in which the partial linker sequence between JmjN and JmjC was deleted (Figure 1B), and examined the histone demethylase activity of each using the assay described above. Similar to full-length JMJ18, the truncated proteins were H3K4-specific demethylases (Figure 1B and Figure S1C). Interestingly, both truncated forms of JMJ18 exhibited H3K4me3 and H3K4me2 demethylase activity, and they were able to convert H3K4me3 to H3K4me2 and H3K4me1 in the presence of H3K4me3 peptide (Figure 1B and Figure S1C). This suggests that the linker between JmjN and JmjC blocks the enzyme's demethylase activity toward H3K4me2 *in vitro*. However, they were unable to demethylate H3K4me2 to H3K3me1 if H3K4me2 peptide was used as the substrate (Figure 1B and Figure S1C).

We further observed that the absence of α-ketoglutarate (α-KG) or presence of EDTA completely abolished the enzyme's demethylase activity (Figure 1C), while a lack of Fe(II) strongly inhibited the enzyme's activity (Figure 1C). Taken together, our results suggest that JMJ18 functions as an H3K4-specific demethylase *in vitro*, and that its activity is dependent on α-KG and Fe(II).

### Characterization of *jmj18* mutants

**Figure 2:**
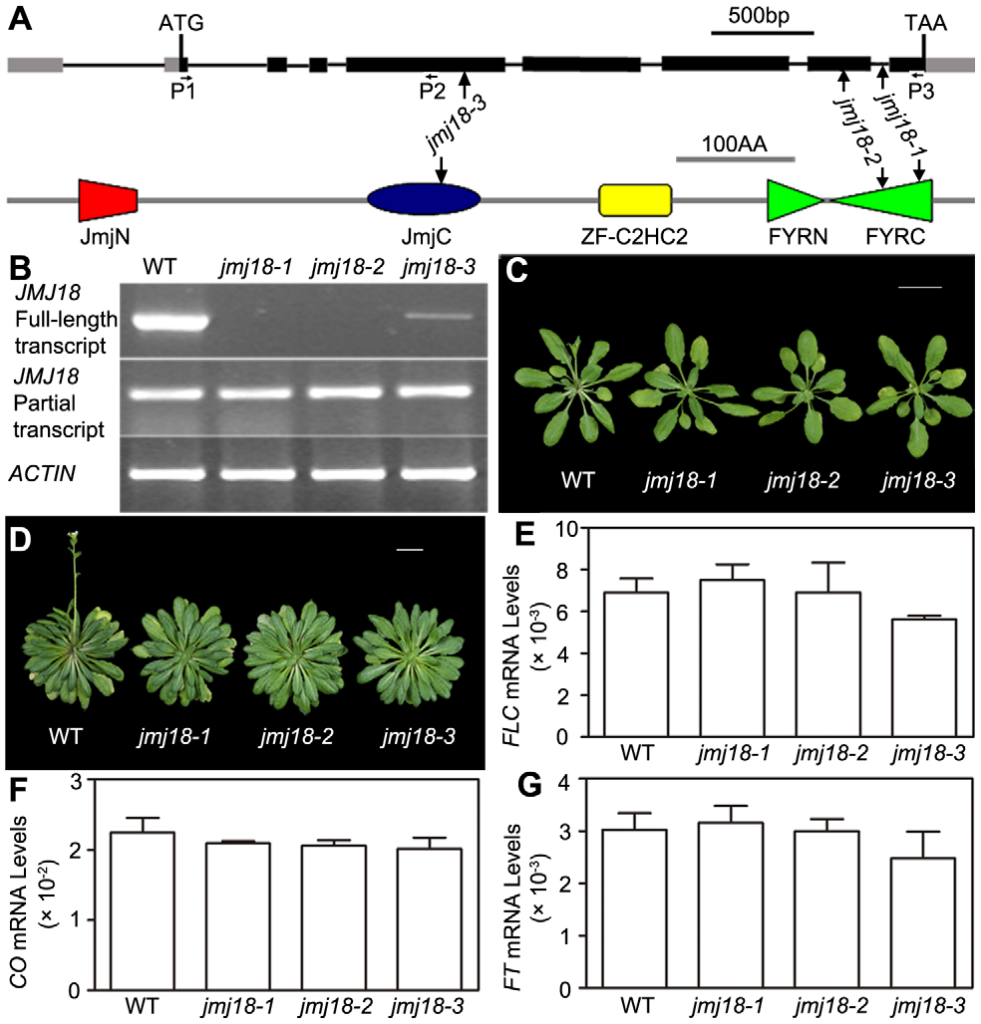
Characterization of three *jmj18* mutants. (A) Structures of *JMJ18* and its gene product. Arrows indicate the T-DNA insertion sites in the mutants. Introns are represented as lines, exons as filled black boxes, and untranslated regions as filled gray boxes. The primers used for transcript detection (P1–P3) are indicated under the schematic gene structure. (B) Transcript analysis of the *jmj18* mutants by RT-PCR. Pairs of P1/P3 and P1/P2 were used for full-length and partial transcripts detection, respectively. The 11-day-old plants of each genotype were collected at dusk for RNA isolation. The *jmj18* mutants exhibited a weak late-flowering phenotype under both LD (C) and SD (D) conditions. Bar = 2 cm. Relative expression levels of *FLC* (E), *CO* (F), and *FT* (G) in the *jmj18* mutants. The expression level was normalized to that of *ACTIN*. RNA was isolated from eleven-day-old seedlings. The values are the mean and standard deviation from three independent experiments. There was no significant difference between wild-type and *jmj18* mutants for the expression levels of *FLC* (E), *CO* (F) or *FT* (G) tested by Student's *t* test (P<0.05).

To assess the biological function of *JMJ18*, we obtained three T-DNA insertion lines from the ABRC [74]: *jmj18-1* (SALK_073442), *jmj18-2* (GABI_649D05), and *jmj18-3* (SAIL_861_F02) (Figure 2A). Reverse transcription (RT)-PCR analysis revealed a lack of full-length *JMJ18* mRNA in *jmj18-1* and *jmj18-2*; however, a partial transcript was detected in both lines (Figure 2B). In the third line, the T-DNA was inserted into exon 4 (Figure 2A), and full-length mRNA was detected at a reduced level compared to that in wild-type plants, and the transcript was confirmed by sequencing, suggesting the existence of a knock-down allele (Figure 2B).

All three alleles exhibited a weak but reproducible late-flowering phenotype under long- (LD) and short-day (SD) conditions (Figure 2C, 2D and Table 1). This late flowering phenotype in *jmj18-1* was complemented by *JMJ18:JMJ18-GFP* transformation (Figure S2). Knock-down lines for *JMJ18* expression by using both double strand RNA interference (RNAi) and artificial micro-RNA (amiR) were generated, and these lines also exhibit weak late-flowering phenotype under LD conditions (Figure S3 and Table S1). To address the molecular mechanism underlying this phenotype, we examined the expression of three important floral regulators, *FLC*, *CONSTANS* (*CO*), and *FT*, by quantitative real-time PCR (qRT-PCR). No obvious change in expression compared to wild-type was detected at various vegetative developmental stages, but statistical significant for the expression of *FLC* and *FT* between wild-type and *jmj18* mutants if any at later vegetative developmental stages (Figure 2E–2G and Figure S4). This suggests that the changes in gene expression are mild, which is consistent with the mild late-flowering phenotype in the *jmj18* mutants and knock-down lines.

**Table 1:** *jmj18* mutants exhibit late-flowering phenotype.

	Genotype	Days to visible buds	Days to first flower open	Rosette leaf no.	Caulin leaf no.	*n*
LD	WT	23.1±2.3	31.8±1.3	11.7±0.9	3.0±0.4	45
	*jmj18-1*	25.9±1.7^*^	33.5±1.4^*^	13.6±1.1^*^	3.1±0.6	45
	*jmj18-2*	26.6±1.5^*^	33.8±1.2^*^	13.5±0.8^*^	2.8±0.6	45
	*jmj18-3*	26.3±2.2^*^	33.8±1.5^*^	13.3±1.1^*^	2.9±0.6	45
SD	WT	70.0±2.6	79.5±2.8	60.7±2.6	8.6±0.5	12
	*jmj18-1*	74.3±2.5^*^	83.3±2.4^*^	65.3±2.4^*^	9.0±0.8	12
	*jmj18-2*	73.4±2.1^*^	82.6±1.5^*^	65.8±2.1^*^	9.6±0.9	12
	*jmj18-3*	73.8±2.3^*^	82.8±3.0^*^	66.2±2.4^*^	9.8±1.1	12

LD, long-day condition (16 h light, 22°C/8 h dark, 18°C, cycle). SD, short-day condition (8 h light, 22°C /16 h dark, 18°C, cycle). The values are the mean ± standard deviation. *n* indicates the plants number scored for phenotype analysis. Asterisks indicate the significant differences in the statistic analysis between WT and mutants using Student's *t* test (P<0.05).

### Spatial expression pattern of *JMJ18*

**Figure 3:**
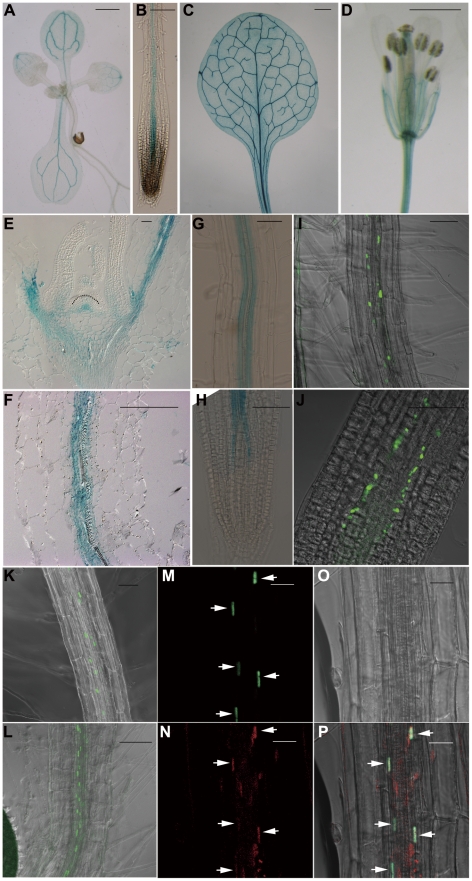
*JMJ18* is expressed predominantly in companion cells. (A–H) Histochemical analysis of *JMJ18_1.4 kb_:GUS* transgenic plants. Ninety-one percent (22 out of 24 independent lines) of the plants exhibited a similar expression pattern. (A) Nine-day-old seedling. (B) Seven-day-old root. (C) Rosette leaves from a 24-day-old plant. (D) Flower. Bar = 1 mm in (A), (C), and (D), and 0.1 mm in (B). Longitudinal sections of the shoot apex (E) and root (F) from twelve-day-old plants. Bar = 50 µm. GUS staining of mature roots (G) and root tips (H) from seven-day-old seedlings. Bar = 50 µm. (I–K) GFP fluorescence in *JMJ18_1.4 kb_:JMJ18-GFP* transgenic plants. Eighty-five percent (17 out of 20 independent lines) of the *JMJ18:JMJ18-GFP* plants displayed a similar expression pattern. Seven-day-old seedlings, mature roots (I) and (K), and root tips (J). Bar = 50 µm. (L) GFP fluorescence in seven-day-old roots of *SUC2:JMJ18-GFP* transgenic plants. Bar = 50 µm. (M) to (P) *JMJ18_1.4 kb_:JMJ18-RFP* and *SUC2:JMJ18-GFP* double-transgenic plants exhibited *JMJ18* expression in companion cells. All of the transgenic lines (13 independent lines) observed for the colocalization of JMJ18-GFP and -RFP displayed a similar colocalization pattern. GFP fluorescence (M), RFP fluorescence (N), bright-field image (O), and merged image (P). Bar = 25 µm.

To gain a deeper understanding of the function of JMJ18 in *Arabidopsis*, we examined its expression pattern using wild-type *Arabidopsis* plants carrying a *JMJ18* promoter-fused GUS reporter construct. The whole intergenic region between *JMJ18* and its upstream gene was first selected to be the promoter of *JMJ18* (Figure S5A). *GUS* expression was detected in vascular tissue collected from the cotyledons, young leaves, and roots of seedlings (Figure 3A and 3B). Similarly, GUS was detected in the vascular tissue of adult leaves and flowers (Figure 3C and 3D). Similar expression pattern was obtained if the length of promoter was increased from 1.4 to 1.7 kb (Figure S5A–S5D).

Next, *JMJ18* expression was examined in detail by microscopy. Longitudinal section analysis showed that *JMJ18:GUS* was expressed in both the shoot and root phloem, as well as in protophloem, but not in the SAM in seedlings (Figure 3E and 3F). Further, *JMJ18* expression was restricted to the cell files of protophloem in the root tip (Figure 3H and Figure S5E–S5J) and phloem companion cells in mature roots (Figure 3F and 3G). These results suggest that *JMJ18* is predominant expressed in companion cells.

The phloem cell-dominant expression of *JMJ18* was further confirmed in transgenic plants expressing GFP-tagged JMJ18 under the control of the *JMJ18* promoter (Figure 3I–3K). *JMJ18-GFP* was expressed in the protophloem in root tip and phloem in other tissues (Figure 3I–3K and Figure S6A–S6C). We also found that JMJ18-GFP was localized to the nucleus (Figure 3I and Figure S6D), which is consistent with its function as a histone demethylase.

To further verify the expression pattern of *JMJ18*, the companion cell-specific *SUC2* promoter [75], [76] was used to drive JMJ18-GFP expression in wild-type plants. The expression pattern of *SUC2:JMJ18-GFP* was similar to that of *JMJ18:JMJ18-GFP* (Figure 3K and 3L). In addition, we generated *JMJ18:JMJ18-RFP* and *SUC2:JMJ18-GFP* double-transgenic plants and found that JMJ18-RFP and JMJ18-GFP were colocalized in the nuclei of companion cells (Figure 3M–3P). It was also observed that JMJ18-GUS was also detected in pollen if the anther was stained longer time (Figure S5K–S5N).These results demonstrate that *JMJ18* is expressed predominant in phloem companion cells, similar to the floral integrator *FT* in vegetative developmental stage [62].

### The temporal expression pattern of *JMJ18* is positively correlated with that of *FT*

**Figure 4:**
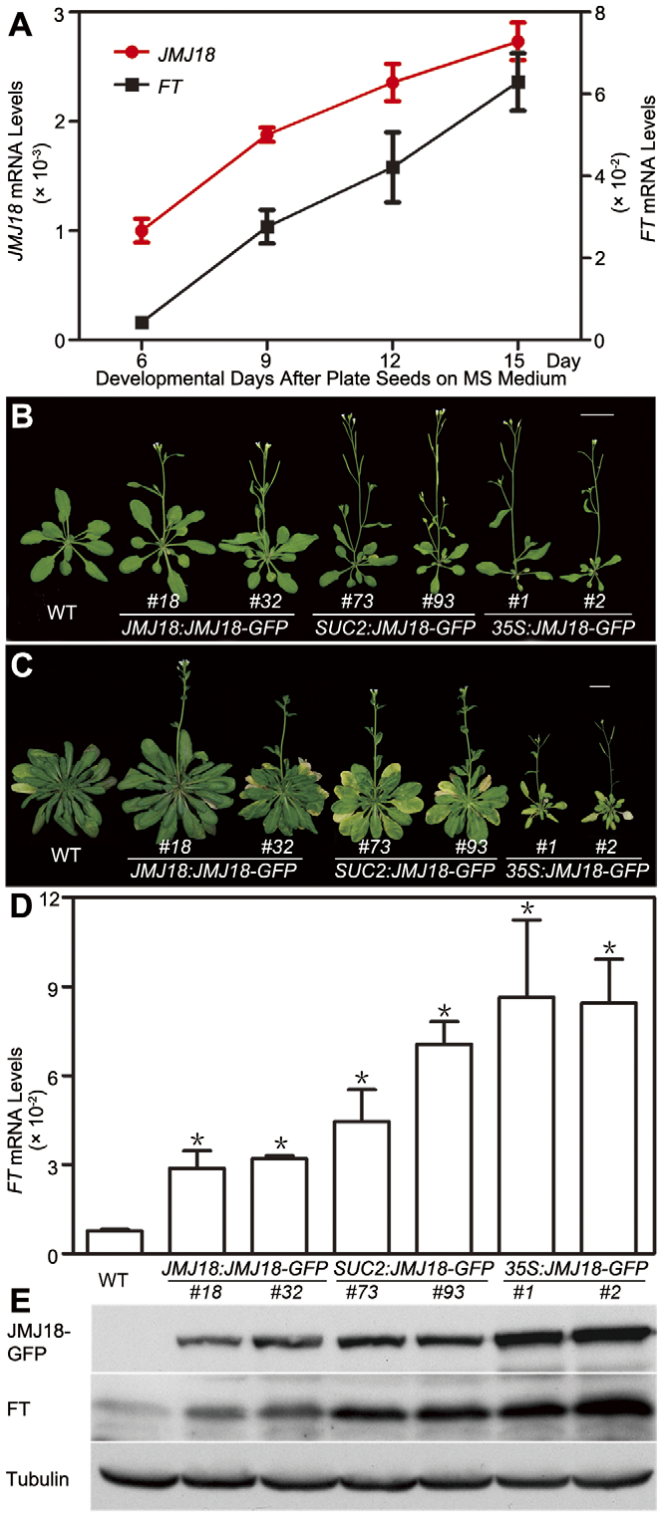
Temporal expression pattern of *JMJ18*, JMJ18-induced *FT* expression, and the floral transition. (A) qRT-PCR analysis of *JMJ18* and *FT* expression in plants grown under LD conditions. The expression level was normalized to that of *ACTIN*. Error bars indicate the standard deviation of three independent biological replicates. (B) JMJ18 overexpression promotes the floral transition under LD conditions. Bar = 2 cm. Twenty-five-day-old plants were analyzed. Twenty-one (23 out of 108), 33 (36 out of 108), and 19 (25 out of 108) percent of the independent lines for *JMJ18:JMJ18-GFP*, *SUC2:JMJ18-GFP*, and *35S:JMJ18-GFP*, respectively, exhibited earlier flowering compared to wild-type plants. The plants selected for phenotypic analysis were homozygous with one insertion at T4 generation for *JMJ18:JMJ18-GFP* and *SUC2:JMJ18-GFP*, and T3 generation for *35S:JMJ18-GFP*. Two independent lines per transformation were shown. (C) *JMJ18* overexpression promotes the floral transition under SD conditions. Bar = 2 cm. Fifty-eight-day-old plants were analyzed. (D) qRT-PCR analysis of *FT* mRNA expression in the transgenic plants. The expression level was normalized to that of *ACTIN*. Error bars indicate the standard deviation of three independent biological replicates. The 11-day-old plants were collected at dusk for the analysis. Asterisks indicate the significant difference between wild-type and overexpression lines using Student's *t* test (P<0.05). (E) Western blot analysis of JMJ18 and FT expression in the transgenic plants. Tubulin was used as a loading control. The 11-day-old plants were harvested at dusk for the analysis.

To determine the developmental expression profiles of *JMJ18*, wild-type seedlings grown under LD conditions were harvested at dusk on days 6, 9, 12, and 15 for RNA isolation. The plants did not form floral meristems; day 15 was the last day before floral meristem formation based on our microscopic observations. *JMJ18* was expressed at a low level in young seedlings; however, its expression progressively increased during vegetative development from day 6 to day 15 (Figure 4A). Thus, the expression of *JMJ18* is developmentally regulated.

*FT* expression was also examined using the same batch of materials. Consistent with previous data [60], [61], *FT* expression was weak in young seedlings; however, it increased steadily as the plants matured (Figure 4A). There was a significant positive correlation between the expression patterns of *JMJ18* and *FT* during vegetative development, with a correlation coefficient of 0.986 (Figure 4A).

### *JMJ18* overexpression promotes flowering through *FT* expression

To further investigate the function of JMJ18, we overexpressed JMJ18 using its endogenous promoter (*JMJ18* promoter), the companion cell-specific *SUC2* promoter, and the constitutively expressed *CaMV35S* promoter of which is expressed in and outside of the companion cells in wild-type plants. All three types of overexpression lines exhibited earlier flowering compared to wild-type under inductive LD and non-inductive SD conditions (Figure 4B, 4C and Table 2). Overall, the *SUC2:JMJ18-GFP* transgenic lines flowered earlier than the *JMJ18:JMJ18-GFP* transgenic plants; however, the *35S:JMJ18-GFP* transgenic lines flowered before either of the other two (Figure 4B, 4C and Table 2). This suggests that the overexpression phenotype was JMJ18 dose-dependent.

**Table 2:** Over-expression of *JMJ18* induces floral transition under long-day and short-day conditions.

	Genotype	Days to visible buds	Days to first flower open	Rosette leaf no.	Cauline leaf no.	*n*
LD	WT	23.9±1.6	31.5±2.0	11.7±1.4	2.2±0.7	30
	*JMJ18:JMJ18-GFP #18*	20.1±0.8	28.0±1.4	9.5±0.7	2.6±0.5	28
	*JMJ18:JMJ18-GFP #32*	18.8±0.8	26.4±0.9	8.9±0.6	2.8±0.4	35
	*SUC2:JMJ18-GFP #73*	18.0±0.5	25.0±0.7	7.0±0.8	3.1±0.4	36
	*SUC:JMJ18-GFP #93*	17.8±0.7	24.8±0.9	6.7±0.7	3.1±0.5	30
	*35S:JMJ18-GFP #1*	18.3±0.8	26.5±0.8	6.0±0.7	2.3±0.5	24
	*35S:JMJ18-GFP #2*	18.3±1.1	25.9±1.4	5.6±0.9	2.2±0.7	26
SD	WT	65.4±2.6	74.6±2.4	57.5±3.3	8.3±0.9	12
	*JMJ18:JMJ18-GFP #18*	59.7±1.8	68.7±1.8	46.6±2.2	7.2±0.7	12
	*JMJ18:JMJ18-GFP #32*	58.3±1.7	67.2±1.5	37.6±2.4	6.8±0.8	12
	*SUC2:JMJ18-GFP #73*	42.9±2.8	51.2±3.4	23.6±2.2	5.6±0.8	12
	*SUC:JMJ18-GFP #93*	45.2±2.2	50.3±2.1	19.8±2.0	4.3±0.8	12
	*35S:JMJ18-GFP #1*	32.3±3.0	42.5±1.8	9.8±1.6	2.3±0.7	12
	*35S:JMJ18-GFP #2*	33.3±4.3	43.0±3.4	9.5±2.0	2.3±0.5	12

LD, long-day condition (16 h light, 22°C/8 h dark, 18°C, cycle). SD, short-day condition (8 h light, 22°C /16 h dark, 18°C, cycle). The length of *JMJ18* promoter is 1.4 kb. Values are the mean number ± standard deviation. *n* indicates the number of plant scored for each line.

To verify this prediction, total protein was extracted from eleven-day-old transgenic plants harvested at dusk, and the abundance of JMJ18 was assessed by Western blotting using anti-JMJ18 antibodies generated against recombinant JMJ18 in rabbits. Consistent with the early-flowering phenotype described above, the expression of JMJ18 in the transgenic plants was as follows (in order from lowest to highest): *JMJ18:JMJ18-GFP*, *SUC2:JMJ18-GFP*, and *35S:JMJ18-GFP* (Figure 4E and Figure S7). Taking our flowering time and JMJ18 expression data together, the overexpression of JMJ18 promotes flowering in a JMJ18 dose-dependent manner.

The strong positive correlation between the *JMJ18* and *FT* spatio-temporal expression patterns (Figure 3, Figure 4A, and Figure S5) suggests that JMJ18 regulates *FT* expression in companion cells to control floral development. To investigate this possibility, total RNA was isolated from the same batch of materials used to examine JMJ18 expression. *FT* expression was increased to varying degrees in all three types of overexpression lines, and the degree of increase in each line was correlated with the abundance of JMJ18 and flowering time (Figure 4D, 4E and Table 2). Similar results were obtained for the expression of *TSF*, a homolog of *FT*, in JMJ18 overexpression lines (Figure S8).

It has been shown that FT is one of the most important components of florigen, and its abundance determines flowering behavior [66], [67], [68]. Thus, we next examined the abundance of FT in JMJ18 overexpression lines by Western blotting using anti-FT antibodies generated against recombinant FT in rabbits. The FT level detected in each line was consistent with the mRNA level and abundance of JMJ18 (Figure 4D and 4E). These results indicate that JMJ18 induces *FT* and its homologs such as *TSF* transcription and enhances their accumulation to accelerate the vegetative-to-reproductive transition.

### The promotion of flowering by JMJ18 mainly depends on FT function

**Figure 5:**
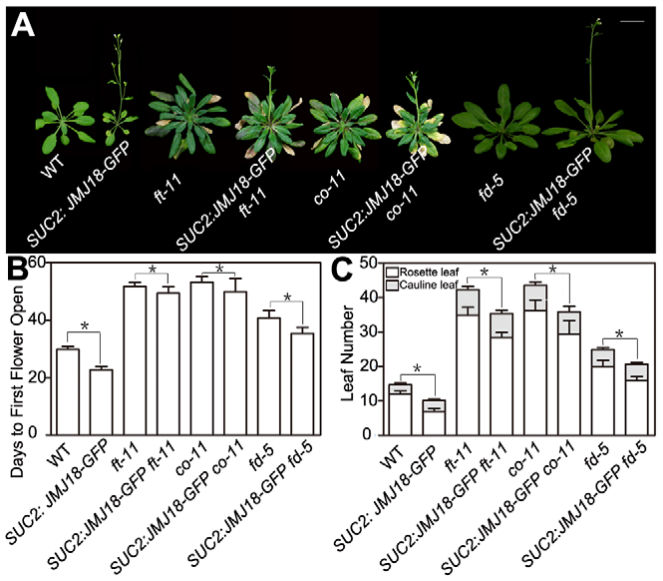
Functional FT is necessary for promotion of the floral transition by JMJ18. Promotion of the floral transition by JMJ18 is mainly dependent on *FT*, *CO*, or *FD*. (A) WT and *SUC2:JMJ18-GFP* are 24-day-old plants; *ft-11*, *SUC2:JMJ18-GFP ft-11*, *co-11*, and *SUC2:JMJ18-GFP co-11* are 43-day-old plants. *fd-5* and *SUC2:JMJ18-GFP fd-5* are 35-day-old plants. *SUC2:JMJ18-GFP* line #73 was used in the experiment. Bar = 2 cm. The values in (B) and (C) are the mean ± standard deviation from at least 20 plants per genotype. Asterisks in (B) and (C) indicate the significant difference between marked plants from the two genotypes by Student's *t* test (P<0.05).

To test whether FT function is genetically necessary for facilitation of the vegetative-to-reproductive transition by JMJ18, *ft-11* was crossed with the *SUC2:JMJ18-GFP* line. The mutation in *FT* dramatically suppressed the early-flowering phenotype of the *SUC2:JMJ18-GFP* plants (Figure 5A–5C), suggesting that *FT* is required for the function of JMJ18 in flowering control.

In the photoperiod pathway, CO is responsible for *FT* induction, and *FT* expression is obviously delayed in a *CO* mutant background even under inductive LD conditions [60], [61]. FD is an SAM-specific transcription factor that interacts with FT in the SAM [64]. Both FT and FD are required for floral meristem formation [64], [65]. The mutation of *CO* or *FD* also abolished the *SUC2:JMJ18-GFP* early-flowering phenotype, such as *SUC2:JMJ18-GFP co-11* or *SUC2:JMJ18-GFP fd-5*, flowered at a similar time to *co-11* or *fd-5* (Figure 5A–5C). In addition, we found that overexpression of *JMJ18* in mutant background, such as *SUC2:JMJ18-GFP ft-11, SUC2:JMJ18-GFP co-11* and *SUC2:JMJ18-GFP fd-5*, flowered slightly earlier than the single mutants *ft-11*, *co-11*, and *fd-5*, in terms of both flowering time and rosette leaf number (Figure 5A–5C). These genetic data demonstrate that promotion of the floral transition by JMJ18 mainly depends on functional FT.

### The JmjN, JmjC, and zinc-finger domains are required for JMJ18 function

**Figure 6:**
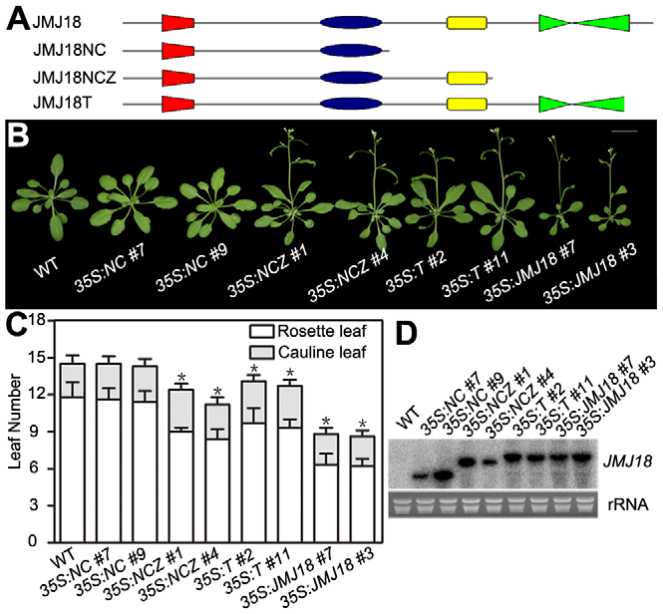
The JmjN, JmjC, and zinc-finger domains are necessary for the promotion of flowering by JMJ18. (A) Schemes display the structures of full-length JMJ18 protein as well as truncated versions used for overexpression test. (B) and (C) Flowering time phenotype for the *JMJ18* overexpressor lines. All of the plants in (B) were grown for 24 days under LD conditions. All of the 108 independent *35S:NC* lines examined flowered at a similar time compared to wild type. Fifteen out of 108 *35S:NCZ*, 20 out of 108 *35S:T*, and 27 out of 108 *35S:JMJ1*8 lines displayed an early-flowering phenotype. Two independent lines per transformation were shown. Bar = 2 cm. The values in (C) are the mean ± standard deviation. At least 15 plants were scored for each genotype. Asterisks indicate the significant difference between wild-type and transgenic plants analyzed by Student's *t* test (P<0.05) using rosette leaf numbers. (D) Northern blot analysis of the expression of truncated *JMJ18* in the transgenic lines.

JMJ18 has four conserved domains: the JmjN, JmjC, zinc-finger, and FY-rich domains (Figure 2A). Due to the obvious early-flowering phenotype of *JMJ18* overexpressors, we overexpressed truncated *JMJ18* (Figure 6A and Figure S9A) in wild-type plants to determine the domain(s) in JMJ18 necessary for its function *in planta*. The early-flowering phenotype of *35S:JMJ18* (containing the entire *JMJ18* CDS) was echoed by that of *35S:JMJ18-GFP* (Figure 6B and 6C), suggesting that blocking of the free of C-terminus of JMJ18 by GFP does not affect its function.

*35S:NCZ* (containing the JmjN, JmjC, and zinc-finger domains but lacking the FY-rich domain) transgenic plants exhibited a shorter life cycle than wild-type plants; however, the effect was weaker than that observed in *35S:JMJ18* plants (Figure 6B and 6C). Regardless, no early-flowering phenotype was observed in those plants overexpressing truncated *JMJ18* with a deletion in both the zinc-finger and FY-rich domains (*35S:NC*) (Figure 6B and 6C). Northern blot analysis was used to determine whether these truncated transcripts were expressed in the transgenic plants. As shown in Figure 6D, all of the truncated transcripts were expressed. The transgenic plants overexpressing truncated *JMJ18* with a deletion in both the JmjN and JmjC domains displayed similar flowering time compared to wild-type plants (Figure S9). Thus, these data suggest that the JmjN, JmjC, and zinc-finger domains are required for the function of JMJ18, while the FY-rich domain is not fully necessary for its function but affects its activity.

The *35S:T* (encoding a truncated protein identical to that in *jmj18-1* and containing the JmjN, JmjC, and zinc-finger domains) transgenic plants also displayed an obviously earlier floral transition compared to wild-type plants (Figure 6A–6D),which indicates that *jmj18-1* and *jmj18-2* are weak alleles. And the result is consistent with their weak early-flowering phenotype (Figure 2C, 2D and Table 1).

### JMJ18 represses the expression of *FLC* and *MAFs*

**Figure 7:**
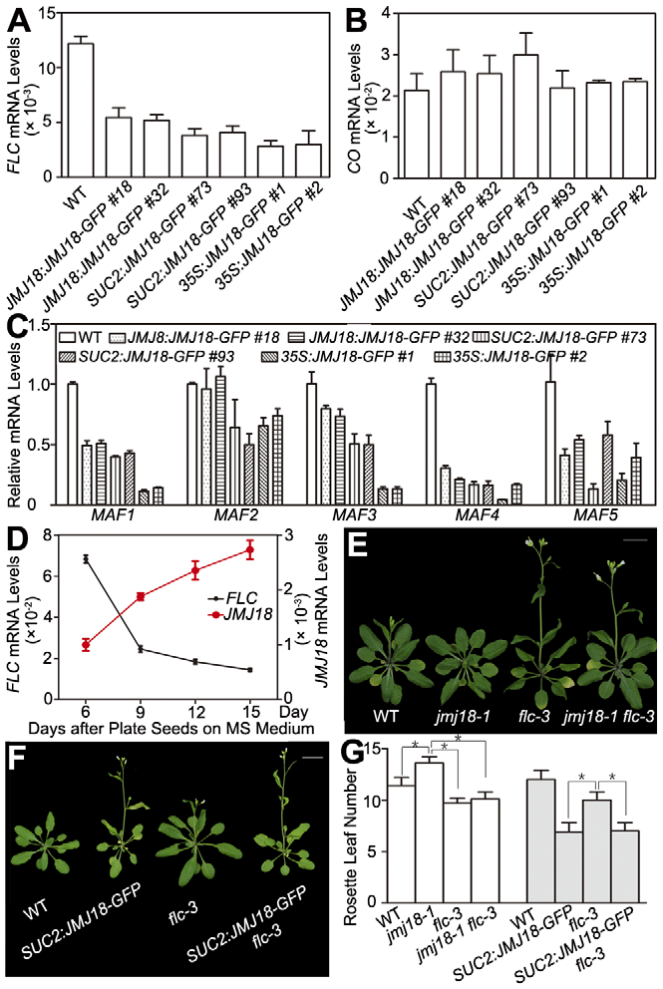
JMJ18 acts as a repressor of *FLC* and *MAFs*. (A) *FLC* expression was repressed in the *JMJ18* overexpressor lines. Asterisks indicate the significant difference between wild-type and transgenic plants analyzed by Student's *t* test (P<0.05). (B) *CO* expression was not obviously changed in the *JMJ18* overexpressor lines. There was no significant difference between wild-type and transgenic plants analyzed by Student's *t* test (P<0.05). (C) The expression of *MAF1* to *MAF5* was repressed in the *JMJ18* overexpressor lines. Eleven-day-old plants were used for RNA isolation. Asterisks indicate the significant difference between wild-type and transgenic plants analyzed by Student's *t* test (P<0.05). The expression level was normalized to that of *ACTIN* in (A) to (C). (D) Temporal expression patterns of *FLC* and *JMJ18* during vegetative development. The mRNA levels were normalized to that of *ACTIN*. The error bars indicate the standard deviation from three independent biological replicates. (E) to (G) Genetic analysis of *JMJ18* and *FLC* in flowering time control. The *SUC2:JMJ18-GFP #73* was crossed to *flc-3*. Twenty-eight- (E) and 24-day-old (F) plants were photographed. Bar = 2 cm. Asterisks indicate the significant difference analyzed by Student's *t* test (P<0.05).

H3K4 methylation is associated with gene activation; thus, H3K4 demethylases should work as gene repressors, rather than as activators. Therefore, *FT* is not likely to be the direct target of JMJ18. The target of JMJ18 could be an upstream repressor of *FT*. If this is the case, the floral repressor *FLC* is a good candidate. To examine this possibility, the effect of JMJ18 overexpression on *FLC* expression was examined in *JMJ18* overexpressor plants. *FLC* expression was significantly repressed in all *JMJ18* overexpressor lines examined, and the degree of repression was positively correlated with the abundance of JMJ18, but negatively with the level of FT (Figure 4E and Figure 7A), the other members of *FLC* clade genes (*MAF1 to MAF5*) were also repressed to different extent in *JMJ18* overexpressors (Figure 7C); in comparison, there was no obvious change in the expression of *CO*, an upstream activator of *FT* (Figure 7B).

To analyze the temporal expression pattern of *FLC* and its relationship to that of *JMJ18* during vegetative development, we measured *FLC* expression in plants grown under LD conditions on days 6, 9, 12, and 15 as described above. *FLC* expression was strong at the seedling stage, then decreased during vegetative development, reaching its lowest level before floral meristem formation on day 15 (Figure 7D). There was a strong negative correlation between the developmental expression patterns of *JMJ18* and *FLC* during vegetative development (r = −0.950). Thus, we propose that JMJ18 is a repressor of *FLC*.

We generated *jmj18-1 flc-3* to test whether *JMJ18* interacts genetically with *FLC*. The *FLC* mutation mainly blocks the late-flowering phenotype of *jmj18-1* (Figure 7E, 7G and Table S2). Thus, the mutation in *FLC* did not enhance the early-flowering phenotype of the *JMJ18* overexpressors. In addition, overexpression of JMJ18 in *flc*, such as *SUC2:JMJ18-GFP flc-3*, flowered earlier than *flc-3* and at almost the same time as *SUC2:JMJ18-GFP*) (Figure 7F, 7G and Table S2), indicating that endogenous JMJ18 enhances flowering mainly by repressing *FLC* in wild-type plants, while overexpressing JMJ18 recognized *FLC* and *MAFs* as targets. Taken together, those results indicate that *JMJ18* and *FLC* belong to the same genetic pathway in flowering time control, and *JMJ18* functions in upstream of *FLC*.

### JMJ18 demethylates the chromatins of *FLC* and *MAFs*

**Figure 8:**
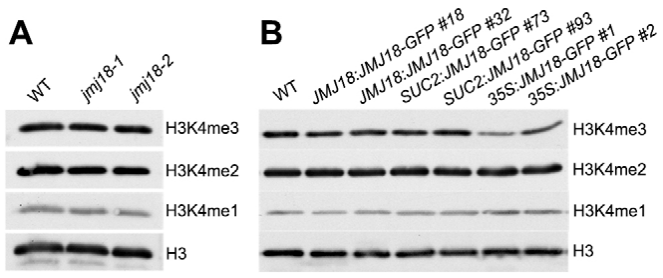
Global H3K4 methylation levels in wild-type, *jmj18*, and *JMJ18* overexpressor lines. (A) Detection of the global H3K4me3, H3K4me2, and H3K4me1 levels in the *jmj18* mutants. (B) Detection of the global H3K4me3, H3K4me2, and H3K4me1 levels in the *JMJ18* overexpressor lines.

Our previous result suggested that JMJ18 functions as an H3K4-specific demethylase *in vitro* (Figure 1 and Figure S1). To verify the histone demethylase activity of JMJ18 *in vivo*, nucleoproteins were extracted from *jmj18*, our *JMJ18* overexpressor lines, and wild-type plants. There was no obvious change in the levels of H3K4me3, H3K4me2, and H3K4me1 among the wild-type, *jmj18*, and *JMJ18* overexpressor plants on a global scale (Figure 8A and 8B). A slight decrease in H3K4me3 was detected in the *35S:JMJ18-GFP* plants (Figure 8B), but no obvious difference was detected between wild-type and the other *JMJ18* overexpressors (Figure 8B). Thus, endogenous JMJ18 may function as a gene-specific H3K4 demethylase *in vivo*, while overexpressed JMJ18 can target many other loci to demethylate H3K4me3 once it is expressed in many cell types.

**Figure 9:**
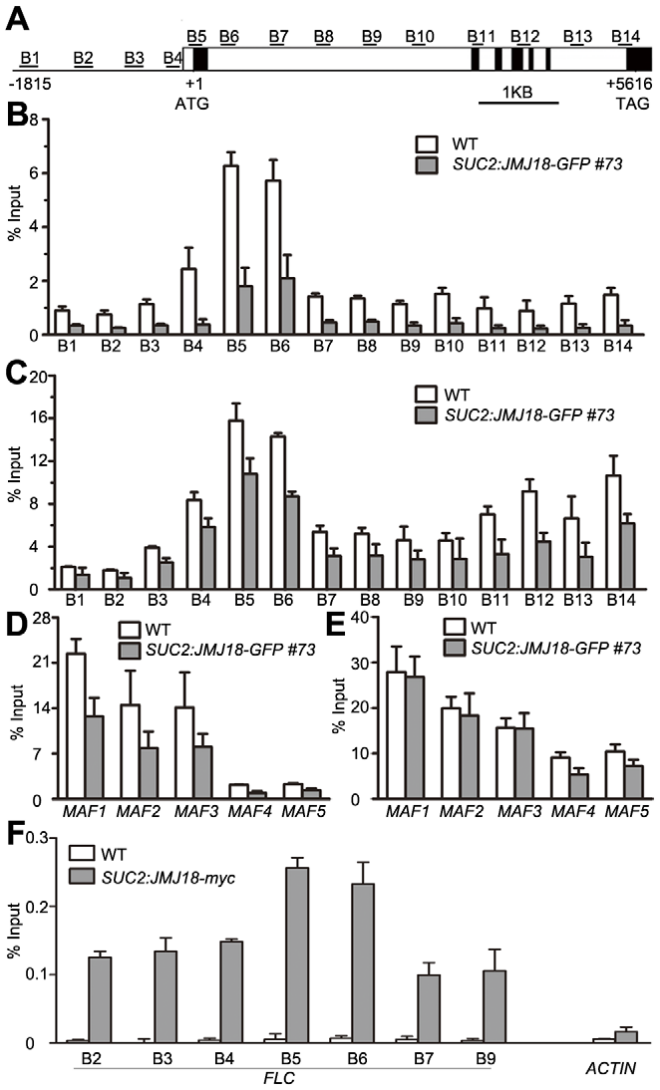
JMJ18 binds *FLC* chromatin and mediates the level of H3K4 methylation in *FLC* chromatin. (A) Schematic genomic structure of the *FLC* locus, and the probes used to measure the histone methylation level. Open boxes represent introns, while filled boxes represent exons. B1–B14 indicate the *FLC* regions recognized by the probes during the analysis of the H3K4 methylation level by ChIP. (B) ChIP analysis of the H3K4me3 level across *FLC* in wild-type and *JMJ18* overexpressor plants. (C) ChIP analysis of the H3K4me2 level across *FLC* in wild-type and *JMJ18* overexpressor plants. (D) and (E) ChIP analysis of the H3K4me3 and H3K4me2 levels for other members of *FLC* clade genes in wild-type and *JMJ18* overexpressor plants. (F) ChIP analysis of the binding of JMJ18 to *FLC* chromatin in eleven-day-old plants. The enrichments were normalized to the input. *ACTIN* was used as a negative control. The values are the mean ± standard deviation from three biological replicates.

To verify whether JMJ18 demethylates *FLC* chromatin, chromatin immunoprecipitation (ChIP) was done to detect the level of H3K4 methylation across the entire *FLC* chromatin region. In wild-type plants, the regions around the transcription start site (regions B4, B5, and B6) had higher levels of H3K4me3 than the other regions (Figure 9A and 9B), which is consistent with previous data [11], [43]. However, the H3K4me3 level was obviously decreased in *SUC2:JMJ18-GFP* plants across the entire *FLC* chromatin region compared to that in wild-type plants (Figure 9B). However, the H3K4me3 levels at the house keeping gene *ACTIN*, which contains high level of H3K4me3, and transposon elements *AtMu1* and *AtSN1*, which are lack of H3K4me3, were not obviously changed in *SUC2:JMJ18-GFP* plants compared to wild-type plants (Figure S10). The similar results were obtained by using normalizing H3K4me3 level to total H3 and to the input (Figure S11). The level of H3K4me2 in *FLC* chromatin was also decreased in *SUC2:JMJ18-GFP* overexpressor plants compared to wild-type plants (Figure 9C). To analyze whether JMJ18 regulates the expression of *MAFs* through the control of the H3K4 methylation state at their loci, the H3K4me3 and me2 modification levels were detected in *SUC2:JMJ18-GFP* and wild-type plants. The levels of H3K4me3 at the chromatins of five *MAFs* were obviously decreased. While, the levels of H3K4me2 were slightly decreased at *MAF4*, *MAF5* but not *MAF1*, *MAF2* and *MAF3* (Figure 9D, 9E). These results indicate that JMJ18 demethylates H3K4me3 and H3K4me2 within the *FLC* and *MAFs* loci *in vivo*.

### JMJ18 directly associates with *FLC* chromatin

JMJ18 controls *FLC* expression by modulating the H3K4 methylation level at *FLC* chromatin, suggesting that JMJ18 associates with the *FLC* locus and mediates the methylation of *FLC* chromatin directly. To investigate this possibility, ChIP was used to detect the binding of JMJ18 to the *FLC* locus. Due to the increased level of nonspecific binding of GFP to *Arabidopsis* chromatin under our experimental conditions, we used *SUC2:JMJ18-myc* transgenic plants instead of *SUC2:JMJ18-GFP* plants in our assay. The *SUC2:JMJ18-myc* transgenic plants exhibited similar phenotypes to the *SUC2:JMJ18-GFP* plants, including an early-flowering phenotype, altered *FLC* and *FT* expression, and reduced H3K4me3 and H3K4me2 levels across *FLC* chromatin (Figure S12). A c-myc-specific antibody was used to precipitate chromatin from wild-type and *SUC2:JMJ18-myc* plants. Compared to the wild-type plants, the *FLC* chromatin of the transgenic plants was significantly enriched with JMJ18 protein, with varying levels of occupancy across the entire *FLC* chromatin region, while, there was no obvious binding of JMJ18 to the chromatin of *ACTIN*, which was a negative control, compared with its binding to *FLC* chromatin (Figure 9F). Regions B5 and B6 exhibited the highest binding ability (Figure 9F), whereas the H3K4 methylation level was higher than in the other regions (Figure 9B and 9C), demonstrating that JMJ18 associates with *FLC* chromatin and that the binding of JMJ18 to *FLC* is necessary for the dynamic of H3K4 methylation in *FLC* chromatin.

### *JMJ18* overexpression suppresses the *FRI* late-flowering phenotype

**Figure 10:**
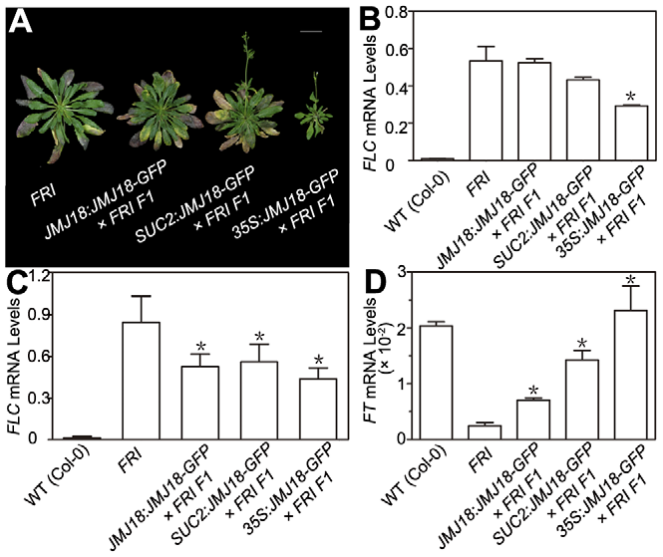
JMJ18 overexpression suppresses the *FRI* late-flowering phenotype. (A) Flowering time in *FRI* wild-type and F1 plants from a cross between *FRI* wild-type and *JMJ18* overexpressor plants. All of the plants were 48 days old at analysis except for *35:JMJ18-GFP #1×FRI F1*, which was 38 days old. All plants were grown under LD conditions. Bar = 2 cm. Expression levels of *FLC* in eleven-day-old whole seedlings (B) and 26-day-old rosette leaves (C). (D) *FT* expression in eleven-day-old seedlings. The expression level was normalized to that of *ACTIN*. The values are the mean and standard deviation from three independent biological replicates. The lines crossed with *FRI* were: *JMJ18:JMJ18-GFP #34*, *SUC2:JMJ18-GFP #73*, and *35S:JMJ18-GFP #1*. Asterisks in (B), (C) and (D) indicate the significant difference for the expression levels of *FLC* and *FT* between *FRI* and other genotypes analyzed by Student's *t* test (P<0.05).

*FRI* is the major determinant of ecotype differences in flowering time in *Arabidopsis* [46], [47]. To examine the influence of JMJ18 on flowering time promotion, JMJ18 was overexpressed in *FRI* plants by the introduction of *FRI* into *JMJ18:JMJ18-GFP*, *SUC2:JMJ18-GFP*, and *35S:JMJ18-GFP* transgenic lines via crossing, respectively. The overexpression of *JMJ18* significantly suppressed the late-flowering phenotype of *FRI* in a JMJ18 dose-dependent manner (Figure 10A and Table 3).

**Table 3:** *JMJ18* overexpression suppresses the *FRI* late-flowering phenotype.

Genotype	Days to visible buds	Days to first flower open	Rosette leaf no.	Cauline leaf no.	*n*
WT (Col-0)	22.2±1.1	29.5±1.4	11.7±1.1	2.8±0.7	29
*FRI*	>90		>80		20
*JMJ18:JMJ18-GFP #32×FRI F1*	48.1±2.9	58.5±3.4	46.7±2.1	7.2±1.0	19
*SUC2:JMJ18-GFP #73×FRI F1*	37.4±2.0	45.7±2.0	38.1±2.0	5.3±1.0	21
*35S:JMJ18-GFP #1×FRI F1*	33.1±4.6	41.4±4.4	15.4±2.8	3.8±0.8	14

The plants were grown under long-day condition. The values are the mean ± standard deviation. *n* indicates the plant number scored for phenotype analysis. The length of *JMJ18* promoter is 1.4 kb.

*FRI* represses the floral transition by activating *FLC* expression [46], [77]. To determine whether JMJ18 suppresses the *FRI* late-flowering phenotype by decreasing *FLC* expression, *FLC* transcription in *FRI* wild-type and *JMJ18* overexpressors was measured. *FLC* expression was decreased in the transgenic lines compared to that in the *FRI* background, with a more pronounced effect in leaves than in complete seedlings except for *35S:JMJ18-GFP* (Figure 10B and 10C), which is consistent with the expression pattern of *FLC* and *JMJ18* in our transgenic lines. This is because *FLC* was expressed in both the SAM and companion cells, with predominant expression in the SAM, while both the *JMJ18* and *SUC2* promoters were specifically expressed in companion cells. Thus, this result supports the notions that JMJ18 promotes flowering in *FRI* by repressing *FLC* expression, and that *FLC* expressed in both the SAM and companion cells contributes to flowering time control. Consistent with our previous results (Figure 4D and Figure S12D) and early-flowering phenotype (Figure 10A and Table 3), the expression of *FT* in these transgenic lines was obviously activated (Figure 10D), indicating that JMJ18 represses *FLC* expression and induces *FT* expression in an *FRI* background as well.

## Discussion

### JMJ18 is a histone H3K4 demethylase in *Arabidopsis*

Several JARID1 family proteins have been characterized as H3K4 demethylases in various organisms [9], [28], [29], [33], [69], [71], [72], [73]. There are 6 members of JARID1 proteins in *Arabidopsis*, JMJ14 (at4g20400), JMJ15 (at2g34880), JMJ16 (at1g08620), JMJ17 (at1g63490), JMJ18 (at1g30810), and JMJ19 (at2g38950). To examine whether *Arabidopsis* JMJ18 is a real histone demethylase, we characterized its histone demethylase activity *in vitro* and *in vivo*. We examined the histone demethylase activity of JMJ18 *in vitro* by purifying recombinant JMJ18 from insect cells and analyzing it by MALDI-TOF mass spectrometry. In that analysis, JMJ18 exhibited histone H3K4-specific demethylase activity (Figure 1A, 1B, and Figure S1), which was dependent on Fe(II) and α-KG (Figure 1C). Additionally, JMJ18 was able to demethylate histone H3K4me3 to H3K4me2 and H3K4me1 (Figure 1A, 1B, and Figures S1C). This observation was confirmed *in vivo* using *FLC* and *MAFs* chromatins (Figure 9B–9E, Figures S11A–11B and S12E–S12F), but not in a genome-wide analysis of histone H3 in *jmj18* mutants or in overexpressing lines driven by *JMJ18-* or *SUC2*- promoters (Figure 8), and the H3K4me3 modification was not obviously changed at *ACTIN*, *AtMu1* and *AtSN1* loci neither (Figures S10 and S11C), suggesting that JMJ18 may function as a gene-specific H3K4 demethylase in *Arabidopsis*. However, it is obvious that global H3K4me3 in *35S:JMJ18-GFP* is reduced (Figure 8B), indicating that JMJ18 can target many other loci to demethylate H3K4me3 but not H3K4me2 once it is expressed in many cell types. Thus, the target specificity of JMJ18 can be fulfilled by gene specific targeting mechanisms or expression restriction or both. It was observed that the reduction in H3K4me2 is less than that H3K4me3 in the chromatins *FLC* and *MAFs* by JMJ18 overexpression (Figure 9B–9E and –S12F), and full-length JMJ18 lacks demethylase activity towards H3K4me2 to H3K4me1 (Figure 1A and Figure S1C), indicating that JMJ18 favors H3K4me3 to H3K4me2 demethylation. Our results also indicate that JARID1 family proteins are conserved histone H3K4 demethylases from human to plants.

Interestingly, full-length JMJ18 only demethylated H3K4me3 to H3K4me2 (Figure 1A). However, truncated JMJ18 lacking the partial linker between the JmjN and JmjC domains exhibited demethylase activity toward both H3K4me3 and H3K4me2 (Figure 1B and Figure S1C). Taken together (Figure 9B–9E and Figure S12E–S12F), our results suggest that the linker between the JmjN and JmjC domains in JMJ18 blocks its H3K4me2 demethylase activity *in vitro*. This linker could not block the H3K4me2 demethylase activity of JMJ18 *in vivo* by functioning as a protein-interaction domain; however, this requires further study. At any rate, this observation raises an interesting question as to the structural function of the linker sequence in JMJ18 itself and within the JMJ18 complex.

### *JMJ18* is a companion cell–predominant, developmentally regulated gene

Cell- or tissue-specific expression is a significant part of enabling a gene to achieve its function in plant cell fate determination and development [64], [65], [78]. Since the transgenic plants carrying *JMJ18:JMJ18-GFP* transformation could complement the late-flowering phenotype of *jmj18-1* (Figure S2), it was possible to characterize the expression pattern of *JMJ18* in *Arabidopsis* based on *JMJ18* promoter-reporter expression (Figure 3, Figures S5 and S6). We found that *JMJ18:GUS* and *JMJ18:GFP* were expressed in the vascular tissue of cotyledons, young leaves, adult leaves, hypocotyl and flowers (Figure 3A, 3C, 3D; Figures S5 and S6), not expressed in the SAM where *FLC* was expressed dominantly (Figure 3E). In the roots of the transgenic plants, signals for GUS and GFP were predominantly observed in protophloem at the root tip, and in phloem in other regions of the root (Figure 3B, 3F–3K; Figures S5 and S6D). In addition, different length of promoter-driven reporter exhibits similar expression pattern (Figure 3, Figures S5 and S6), indicating that JMJ18 is predominantly expressed in the phloem of vascular tissue during vegetative development. Recently, Hong et al. (2009) reported that the expression of *JMJ18* (*Atjmj8* in their study) is expressed broader in root and leaf [79]. We constructed a same version of *JMJ18 promoter-GUS* fusion as Hong *et al* (2009) and transformed it into the wild-type plants. We observed that all 21 independent lines transformed with 1.7 kb length promoter-GUS we examined exhibits similar expression pattern, and the two versions of promoter (1.4 and 1.7 kb in length) exhibit very similar expression patterns (Figure 3, Figures S5 and S6). One possibility for the discrepancy between the two studies is due to the over-staining of GUS of tissues in study of Hong *et al.* (2009) [79].

Phloem is composed of sieve elements and companion cells. An analysis of the cell-specific expression pattern of *JMJ18:GFP* in phloem indicated that *JMJ18* is predominantly expressed in phloem companion cells (Figure 3I, 3K, and Figure S5). This result was confirmed by examining the cellular localization of tagged JMJ18 in the phloem using *Arabidopsis* coexpressing *JMJ18:JMJ18-RFP* and *SUC2:JMJ18-GFP*. *SUC2* is a companion cell-specific gene in *Arabidopsis* [76], [80]. JMJ18-RFP and -GFP were colocalized in the same cell in the roots of the transgenic plants (Figure 3M–3P), indicating that *JMJ18* is predominantly expressed in companion cells. Thus, our results reveal that JMJ18 is a companion cell-dominant histone H3K4 demethylase in *Arabidopsis*.

In addition, the expression of *JMJ18* increased during vegetative development, reaching its high level before the formation of the floral meristem (Figure 4A). This suggests that *JMJ18* encodes a companion cell-dominant, developmentally-regulated histone demethylase in *Arabidopsis*.

### JMJ18 regulates flowering time

To determine the role of JMJ18 in *Arabidopsis* development, we characterized three *jmj18* T-DNA insertion mutants. All three mutants displayed a late-flowering phenotype under LD and SD conditions (Figure 2C, 2D and Table 1). The knock-down lines for *JMJ18* using both RNAi and amiR also exhibit delayed flowering (Figure S3 and Table S1). The late-flowering phenotype in *jmj18* is complemented by transforming *JMJ18:JMJ18-GFP* to *jmj18* (Figure S2). However, the flowering phenotype was weak, and the changes in the expression of flowering marker genes in *jmj18* were very mild (Figure 2E–2G, Figures S3 and S4).

To determine the reason for the weak phenotype of *jmj18*, we further characterized the three *jmj18* alleles. We found that the T-DNA was inserted at the 3′-end of *JMJ18*, behind the JmjN, JmjC, and zinc-finger domains, in both *jmj18-1* and *jmj18-2* (Figure 2A). Partial transcripts of *JMJ18* were detected in *jmj18-1* and *jmj18-2* (Figure 2B). In addition, the truncated *JMJ18* in *jmj18-1* and *jmj18-2* was functional in *planta* (Figure 6B and 6C). For the third allele, although the T-DNA was inserted in the fourth exon of *JMJ18*, full-length *JMJ18* mRNA was detected at reduced levels in *jmj18-3* (Figure 2B). These results indicate that the three *jmj18* alleles were weak mutants. Unfortunately, no other allele for *jmj18* is currently available.

However, the increase in JMJ18 activity caused by the overexpression of *JMJ18* significantly enhanced flowering in *Arabidopsis* (Figure 4B, 4C; Figure 6B, 6C; Figure 10A; Table 2 and Table 3; and ). This observation was true for all *JMJ18* overexpressors driven by the constitutive *CaMV35S* promoter, companion cell-specific *SUC2* promoter, and endogenous *JMJ18* promoter (Figure 4B, 4C; Figure 6B, 6C; Figure 10A; Table 2 and Table 3; and Figure S12A, S12B). The above data suggest that JMJ18 is a flowering time regulator in *Arabidopsis*.

### JMJ18 directly represses *FLC* expression and indirectly induces *FT* expression in companion cells to control flowering time in *Arabidopsis*

FLC is a central repressor of flowering in *Arabidopsis* that works in part through the repression of a companion cell-specific flowering activator, *FT* [55]. *FLC* is expressed in both the SAM and companion cells, and the expressed *FLC* in both tissues contributes to flowering time control in *Arabidopsis* [55]. The regulation of *FLC* expression in the SAM has been extensively studied [11], [24], [43], [53]; however, little is known about the regulation of *FLC* expression in companion cells.

In the present work, we found that *JMJ18* was predominantly expressed in companion cells in vegetative tissue (Figure 3, Figures S5 and S6). In addition, we confirmed that the expression pattern of *JMJ18* was strongly negatively correlated with that of *FLC*, but strongly positively correlated with that of *FT*, during vegetative development in *Arabidopsis* (Figure 4A and Figure 7D). These results suggest that JMJ18 works as an endogenous developmental signal to regulate the expression of *FLC* and *FT* in the control of flowering time.

We further found that JMJ18 binds *FLC* chromatin (Figure 9F), and decreases the levels of H3K4me3 and H3K4m2 in *FLC* chromatin (Figure 9B, 9C; Figures S11A and S12E–S12F). In addition, an increase in JMJ18 abundance obviously decreased the mRNA levels of *FLC* and *MAFs* (Figure 4E; Figure 7A, 7C, 7D; Figure 10B, 10C; and Figure S12C), and obviously increased the expression of *FT* at both the mRNA and protein levels (Figure 4D, 4E; Figure 10D; Figure S12D). Furthermore, the promotion of flowering time by JMJ18 was mainly dependent on functional FT (Figure 5A–5C), but was not enhanced by mutation in *FLC* (Figure 7F, 7G, and Table 2). These results indicate that JMJ18 works as an endogenous, companion cell-dominant developmental signal that directly represses *FLC* expression and indirectly induces *FT* expression in companion cells to control flowering time during vegetative development in *Arabidopsis*. It was noted that overexpressed JMJ18-GFP in companion cells reduced both levels of H3K4me3 and H3K4me2 on *FLC*, but only H3K4me3 on *MAFs* (Figure 9B–9E and Figure S12E–S12F). The difference of the decrease in the levels of H3K4 methylation between *FLC* and *MAFs* in JMJ18 overexpression line may suggest that JMJ18 is more important for the regulation of *FLC* than *MAFs* in *Arabidopsis*.

In *Arabidopsis*, the flowering transition is a complicated process. It needs to integrate the environmental cues and internal signals; in addition, these signals will be sensed and regulated in different tissues or organs in the plant [81], [82]. There are two main tissues involved in the flowering control: the vascular tissue in the leaf and the SAM [37], [83]. The character of the apical meristem is determined not only by the processes occurring in apical meristem, but also by the signals transmitted from vascular tissue. Thus, different flowering regulators should be functional and regulated in distinct tissues or steps, integrated in the SAM for the precise control of flowering time. As flowering regulators, the JmjC domain-containing demethylases are functional in both vascular tissue and SAM, and target different genes. Since REF6 is characterized as a H3K27 demethylase and represses *FLC* in both SAM and leaves, *FLC* can not be the direct target of REF6 [31], [70]. However, its homolog ELF6, an H3K4 demethylase, is expressed in leaves and targets *FT* to repress flowering [28]. Another H3K4 demethylase, JMJ14, is restricted in leaves to repress flowering by decreasing the expression of the floral integrators including *FT*, *SOC1*, *LFY* and *AP1* [28], [29], [69]. In this report, we demonstrated that JMJ18 is a H3K4 demethylase and specially expressed in the vascular tissue (Figure 1, Figure 3, Figure 9; Figures S5, S6, S11, and S12E–S12F). The expression of *JMJ18* is developmental-regulated (Figure 4A). JMJ18 directly represses the expression of *FLC* and releases the expression of *FT* in vascular tissue (Figure 4, Figure 5, Figure 7, Figure 9, Figure 10, and Figure S12), then the released FT is transmitted from the vascular tissue to the SAM to stimulating flowering. These results suggest that plants may have evolved to the point where they use a family of proteins with different expression patterns which target various genes to integrate environmental cues and internal signals to precisely control flowering time. Thus, this study provides novel insight into the regulation of *FLC* expression in companion cells and the epigenetic control of the floral transition in *Arabidopsis*.

## Methods

### Plant materials and growth conditions

The *Arabidopsis* plants used in our experiments were of the Columbia-0 ecotype except for *FRI*. The seeds were first sterilized with 2.25% bleach, then washed three times with water, stratified for three days at 4°C, and then put on Murashige and Skoog (MS) medium (Sigma-Aldrich) containing 1% sucrose and 0.3% phytagel (Sigma-Aldrich). Following ten days of growth under LD (16 h of light, 22°C/8 h of dark, 18°C) or SD (8 h of light, 22°C /16 h of dark, 18°C) conditions in a growth chamber (Percival CU36L5) under a cool white fluorescent light (160 µmolm^−2^ s^−1^), the plants were transplanted to soil and grown in a growth room under LD (16 h of light, 22°C/8 h of dark, 18°C) or SD (8 h of light, 22°C/6 h of dark, 18°C) conditions at 50% relative humidity.

*jmj18-1* (SALK_073442), *jmj18-2* (GABI-649D05), *jmj18-3* (SAIL-861-F02), *ft-11* (GABI-290E08), and *co-11* (SAIL_24_H04) were obtained from the ABRC. *flc-3*, *fd-5*, and *FRI*-Col were described previously [44], [64], [84]. All mutations were confirmed by PCR and sequencing.

### Plant transformation

To construct *JMJ18:GUS*, 1,361 and 1667-bp genomic sequence upstream of the *JMJ18* ATG were PCR-amplified and fused with *GUS*. The primers used for the two versions of promoter were forward for 1,361 bp 5′-CTGCAGACAAGACAGAAGAAGCAGGAAAC-3′, 1667 bp 5′-CTGCAGTGCGAGTTCTTCTTTCAATGTG-3′ and reverse for both, 5′-TCTAGAATGAATTGAAAAATCAATACTACTCAA-3′. The *SUC2* promoter was cloned as described previously [76], [80] using the primers *SUC2* promoter forward 5′-CTGCAGAAAATCTGGTTTCATATTAATTTCA-3′, and reverse 5′-TCTAGAATTTGACAAACCAAGAAAGTAAGA-3′.

We used the following primers to amplify the full-length *JMJ18* CDS: *JMJ18* forward 5′-TCTAGAATGGAAAATCCTCCATTAGAATC-3′, and reverse, 5′-GGATCCCCATCAAATCTACTCCGAAAAGTT-3′. *GFP*, *RFP*, or 6×c-myc was fused to the C-terminus of the *JMJ18* CDS to produce tagged *JMJ18*. To construct *35S:NC*, *35S:NCZ*, *35S:T*, *35S:JMJ18* and *35S:ZY*, the following primers were used to amplify the truncated or full-length *JMJ18* CDS: *JMJ18* forward 5′-TCTAGAATGGAAAATCCTCCATTAGAATC-3′; *JMJ18 NC* reverse 5′-GAGCTCTTAATATAGTTCCACAGCGTTCTGTC-3′; *JMJ18 NCZ* reverse GAGCTCTTAACCTTCCTTTAACTTCTTCTCCTC-3′; *JMJ18 T* reverse, 5′-GAGCTCTTAAACTATAGAGGGTGAGAGGAAACC-3′; *JMJ ZY* forward, 5′-TCTAGAATGCAGAACGCTGTGGAACTATATAG-3′ and *JMJ18* CDS reverse 5′-GAGCTCTTACATCAAATCTACTCCGAAAAGTT-3′.

For plant transformation, the constructs were transformed into *Agrobacterium tumefaciens* strain GV3101. Plants were transformed using the floral dip method [85]. Transformants were selected on MS medium containing kanamycin or hygromycin. Single insertion lines were selected based on the segregation of antibiotic resistance.

### JMJ18 purification and histone demethylase activity assay

Full-length and truncated (*JMJ18Δ420–432 bp* and *JMJ18Δ420–474 bp*) versions of *JMJ18* were generated by PCR amplification from *Arabidopsis* cDNA and cloned into the pFastBac expression vector (Invitrogen). Baculoviruses expressing the various proteins were generated by transfecting the appropriate vector into High5 insect cells. The proteins were purified using Ni-NTA affinity resin (Qiagen). Select fractions were concentrated and purified by size-exclusion chromatography.

The histone demethylase assay and MALDI-TOF mass spectrometry were performed as described previously [21]. About 2 µg of purified full-length or truncated recombinant JMJ18 were incubated with 10 µM peptides at 37°C for 3 h in histone demethylase reaction buffer (50 mM Tris-HCl [pH 7.5], 150 mM NaCl, 50 µM [NH4]_2_Fe[SO_4_]_2_, 2 mM ascorbic acid, and 1 mM α-KG). The reaction was stopped by freezing, and the mixture was diluted for mass spectrometry. The peptides used were: H3K4me3 ARTK(me3)QTARKS, H3K4me2 ARTK(me2)QTARKS, H3K36me3 ATGGVK(me3)KPHRYR, H3K36me2 ATGGVK(me2)KPHRYR, H3K9me3 ARTKQTARK(me3)STGGKAPRAQ, H3K9me2 ARTKQTARK(me2)STGGKAY, H3K27me3 ATKAARK(me3)SAPATG, and H3K27me2 ATKAARK(me2)SAPATG.

### Histological and cytological analyses of *JMJ18* expression

GUS staining was performed as described previously [86], [87]. Twenty-four independent *JMJ18:GUS* T2 lines were used for histochemical analysis, with at least ten individual plants observed for each line. Pictures were taken using a stereomicroscope (Leica MZ16) or light microscope (Zeiss Imager M1).

For the observation of GFP fluorescence, roots were cut from seedlings grown on MS medium under LD conditions for seven days. For *JMJ18:JMJ18-GFP*, 20 independent lines were observed, with at least ten individual plants analyzed per line. Images were collected using a Zeiss LSM 510 Meta confocal laser scanning microscope as described previously [80].

### Observation of the colocalization of JMJ18-GFP and -RFP

*JMJ18:JMJ18-RFP* was constructed to pCAMBIA2300 and introduced into *SUC2:JMJ18-GFP* (in pCAMBIA1300) transgenic plants. The double transformants were selected on MS medium containing kanamycin and hygromycin. Transgenic lines expressing JMJ18-GFP and -RFP were selected and photographed using a Zeiss LSM 510 Meta confocal laser scanning microscope.

### Gene expression analysis

Total RNA was isolated from the plant materials indicated in the text using RNAiso plus (Takara) and treated with RNA-free DNase (Promega) to remove all remaining DNA. Three micrograms of total RNA were used to synthesize first-strand cDNA with a reverse transcription kit (Fermentas). The cDNAs were diluted to 60 µl with sterilized water. One microliter of diluted cDNA was used for real-time PCR amplification with SYBR Premix Ex Taq (Takara). Three biological replicates were performed to calculate the mRNA abundance. Standard deviations were calculated from the replicates. The primers used were listed in Table S3.

### ChIP assay

Plant tissue (1.5 g) was collected after growth under LD conditions for eleven days. ChIP was performed as described previously with three biological replicates [88], [89]. The results are shown as absolute enrichment compared to the input or to total H3. The antibodies used were: anti-H3K4me3 (Upstate 04-745), -H3K4me2 (Upstate 07-030), and -c-myc (Sigma-Aldrich M4439). The primers used to measure the amount of DNA from the ChIP products were listed in Table S4.

### Preparation of anti-JMJ18 and -FT antibodies

Purified recombinant JMJ18 from insect cells and FT from *E. coli* were used as antigens to immunize rabbits. Each rabbit was immunized once every fourteen days with 1 mg of protein. After four successive immunizations, the anti-serum was examined by ELISA and Western blotting using total protein from the mutant and overexpressor lines.

### Western blot analysis

For immunoblotting, *Arabidopsis* seedlings were ground to a powder in liquid nitrogen then homogenized in extraction buffer (10 mM Tris-HCl [pH 7.5], 150 mM NaCl, 10 mM MgCl_2_, 1% Nonidet P-40, complete protease inhibitor [Roche], and 1 mM phenylmethylsulfonyl fluoride). The extracts were then centrifuged, the pellet removed, and the supernatant boiled in 6× SDS sample buffer. The proteins in the samples were separated by 8% SDS-PAGE, transferred to polyvinylidene difluoride membranes (Millipore), and detected using different antibodies. The antibody used for Tubulin detection was anti-α-Tubulin (Sigma-Aldrich T5168).

### Double-strand RNA and amiRNA interferences

To knock-down the expression of *JMJ18*, a double strand RNA and artificial microRNA interference approaches were used. For a double strand RNA RNAi, a unique 616 bp *JMJ18* sequence was amplified by PCR. The gene-specific primers were: forward 5′-GGGGTACCTCTAGAGGTGAAGGCTCTTTGGGAACTC-3′, reverse 5′-CGGGATCCGAGCTCCTCGACCGATACACCAAGGTTC-3′. The self-complementary hairpin RNA was constructed to pTCK303 as described by Wesley *et al.* (2001) [90].

Three primer pairs were designed through the web (http://wmd2.weigelworld.org/cgi-bin/mirnatools.pl?page=1) for artificial microRNA interference assay. The primers are: amiR-s, 5′-GATGAGTCTTTAAAATGCAGGGCTCTCTCTTTTGTATTCC-3′, amiR-a, 5′-GAGCCCTGCATTTTAAAGACTCATCAAAGAGAATCAATGA-3′, amiR*s, 5′-GAGCACTGCATTTTATAGACTCTTCACAGGTCGTGATATG-3′, amiR*a, 5′-GAAGAGTCTATAAAATGCAGTGCTCTACATATATATTCCT-3′. The constructs for amiRNAi were established by following the protocols from Schwab *et al.*(Schwab et al., 2006). The resulting three constructs were delivered to wild-type through agrobacterium–mediated transformation to generate *JMJ18* amiRNA interference lines.

### Accession numbers

Sequence data from this article can be found in the *Arabidopsis* Genome Initiative database under the following accession numbers: *ACTIN* (At5g09810), *CO* (At5g15840), *FLC* (At5g10140), *FT* (At1g65480), *FRI* (At4g00650), *JMJ18* (At1g30810), *MAF1* (At1g77080), *MAF2* (At5g65050), *MAF3* (At5g65060), *MAF4* (At5g65070), *MAF5* (At5g65080), *SUC2* (At1g22710) and *TSF* (At4g20370).

## Supporting Information

Figure S1 His-JMJ18 purification and characterization of its histone demethylase activity. (A) SDS-PAGE of purified recombinant His-JMJ18 from High5 insect cells. M, molecular standard; Total, total lysate; Input, cleared input. F1–F5, the fractions eluted from the nickel-affinity column. The numbers represent the molecular weights. (B) Purification of His-JMJ18 by size-exclusion chromatography. Top panel, UV absorbance; Bottom panel, results of the SDS-PAGE analysis of the purified and eluted recombinant His-JMJ18 fractions produced by size-exclusion chromatography. (C) Summary of the histone demethylase activity of His-JMJ18 *in vitro*.
(TIF)

Figure S2 Complementation assay of *jmj18-1* by *JMJ18:JMJ18-GFP* transformation. (A) The late-flowering phenotype of *jmj18-1* was complemented by *JMJ18:JMJ18-GFP* transformation. Twenty-eight-day-old plants were photographed. Bar = 2 cm. (B) and (C) Statistical analysis the phenotype of complementary plants by days to first flower open (B) and rosettle leaf number (C). At least 16 plants of each genotype were used for analysis. Asterisks indicate the significant differences analyzed by Student's *t* test (P<0.05).
(TIF)

Figure S3 Characterization of *JMJ18* RNAi and amiR lines. The gene expression levels of *JMJ18*, *FLC* and *FT* in *JMJ18* knock-down transgenic lines. RNAi: knock-down the expression of *JMJ18* by double strands of RNA. amiR: knock-down the expression of *JMJ18* by artificial microRNA. The numbers in parentheses indicate the amplification cycles.
(TIF)

Figure S4 The expression levels of *FLC* and *FT* in *jmj18* mutants at differential vegetative developmental stages. The expression levels of *FLC* at day 6 (A), 9 (C) and 15 (E), and *FT* at day 6 (B), 9 (D) and 15 (F) grown under long-day condition were measured. The eleven-day-old seedlings were collected at dusk at the day indicated. The expression level was normalized to that of *ACTIN*. Error bars indicate the standard deviation of three replicates. Asterisks indicate the significant difference between wild-type and *jmj18* mutants analyzed by Student's *t* test (P<0.05).
(TIF)

Figure S5 The expression pattern of *JMJ18*. (A) Two promoter regions selected for GUS constructs. The black filled boxes are shown exons, black lines for introns, and gray lines for intergenic sequence. (B) 7-day-old cotyledon. Bar = 1 mm. (C) 20-day-old rosette leaf. Bar = 1 mm. (D) 10-day-old root. Bar = 0.5 cm. (E)–(J) The marked regions of the root in (D). Bar = 0.1 mm. GUS staining in anther (K, M) and (L, N). 21 independent T2 lines were used for GUS staining. *JMJ18_1.7 kb_:GUS* transgenic plants were used in (A)–(L). All 21 independent lines we examined display the similar expression pattern for *JMJ18*. *JMJ18_1.4 kb_:GUS* transgenic plants were used in (M) and (N).
(TIF)

Figure S6 The expression pattern of *JMJ18* in stem, hypocotyl and petal by using *JMJ18_1.4 kb_:JMJ18-GFP* and nuclear localization of JMJ18. *JMJ18* was expressed in companion cells in different tissues. GFP fluorescence was detected in (A) the junction region between cotyledon and hypocotyl from 7-day-old seedling, where the vascular tissue branched, bar = 100 µm; (B) 7-day-old hypocotyl, bar = 50 µm and (C) petal, bar = 20 µm, respectively. (D) Nuclear localization of JMJ18. Bar = 20 µm.
(TIF)

Figure S7 JMJ18 in JMJ18 overexpression and wild-type plants. The *35:JMJ18* transgenic plant was used to mark JMJ18 band, about 20% amount of the other samples was loaded for the sample from *35:JMJ18* transgenic plant. The arrows indicated the JMJ18-GFP or JMJ18 bands. The upper panel: the gel was exposure for 1 min; the lower panel: the gel was exposure for 20 min. Tubulin was used as a loading control.
(TIF)

Figure S8 JMJ18 induced *TSF* expression. qRT-PCR analysis of *TSF* mRNA expression in the transgenic plants. The expression level was normalized to that of *ACTIN*. Error bars indicate the standard deviation of three independent biological replicates. Asterisks indicate the significant difference between wild-type and transgenic plants analyzed by Student's *t* test (P<0.05).
(TIF)

Figure S9 Characterizing *35S:ZY* transgenic plants. (A) Schematic structures of full-length JMJ18 protein as well as JMJ18 ZY. (B) Flowering time distributions of wild-type and *35S:ZY* at T1 generation measured by rosette leaf number. Thirty-two wild-type and 108 independent transgenic plants were analyzed. (C) Flowering time of wild-type and *35S:ZY* transgenic plants counted by rosette leaf number. (D) *JMJ18 ZY* transcription levels in independent *35S:ZY* transgenic line, and the rosette leaf number for each plant was indicated.
(TIF)

Figure S10 JMJ18 do not affect the H3K4me3 modification levels at *ACTIN*, *AtMu1* and *AtSN1* loci. The H3K4me3 modification levels were detected in wild-type and *SUC2:JMJ18-GFP* plants. The results were normalized to the input. The values are the mean ± standard deviation from three biological replicates. Asterisk indicates the significant difference between wild-type and transgenic plants analyzed by Student's *t* test (P<0.05).
(TIF)

Figure S11 JMJ18 decreases *FLC/MAFs* H3K4me3 levels. (A) Overexpression *JMJ18* reduced H3K4me3 level at *FLC* locus in *JMJ18* overexpression plant compared to wild-type plant. (B) ChIP analysis of H3K4me3 levels for other members of *FLC* clade genes. (C) The H3K4me3 levels were not obviously changed at *ACTIN*, *AtMu1* or *AtSN1* chromatin between wild-type and *JMJ18* overexpression plants. The enrichments were normalized to total H3. The values are the mean ± standard deviation from three biological replicates.
(TIF)

Figure S12 Early-flowering phenotype of the *SUC2:JMJ18-myc* line. (A) Twenty-four-day-old *SUC2:JMJ18-myc* plants grown under LD conditions were shown. Bar = 2 cm. Thirty-three out of 108 independent *SUC2:JMJ18-myc* lines displayed the early-flowering phenotype. The independent line shown was used to analyze *FLC* and *FT* expression and for ChIP. (B) Flowering time in wild-type plants and the *SUC2:JMJ18-myc* line based on the number of rosette leaves at flowering. The values are the mean ± standard deviation from 20 plants. qRT-PCR analysis of *FLC* (C) and *FT* (D) expression. The expression level was normalized to that of *ACTIN*. The levels of H3K4me3 (E) and H3K4me2 (F) across the *FLC* genomic region in wild-type and *SUC2:JMJ18-myc* plants were determined. The values in (C) to (F) are the mean and standard deviation from three technical replicates.
(TIF)

Table S1 Knock-down *JMJ18* expression leads to late-flowering phenotype. All the plants were grown in LD condition. The values are the mean ± standard deviation. *n* indicates the plants number scored for the analysis. Asterisks indicate the significant differences in the statistic analysis between wide type and mutants using Student's *t* test (P<0.05).
(DOC)

Table S2 Genetic interaction between *JMJ18* and *FLC* in flowering time control. All the plants were grown under long-day condition. The values are the mean ± standard deviation. *n* indicates the plant number scored for phenotype analysis.
(DOC)

Table S3 The primers used for detecting gene expression level.
(DOC)

Table S4 The primers used in ChIP assay.
(DOC)

## References

[1] Luger K, Mader AW, Richmond RK, Sargent DF, Richmond TJ (1997). Crystal structure of the nucleosome core particle at 2.8 A resolution.. Nature.

[2] Kouzarides T (2007). Chromatin modifications and their function.. Cell.

[3] Li B, Carey M, Workman JL (2007). The role of chromatin during transcription.. Cell.

[4] Bannister AJ, Zegerman P, Partridge JF, Miska EA, Thomas JO (2001). Selective recognition of methylated lysine 9 on histone H3 by the HP1 chromo domain.. Nature.

[5] Kim T, Buratowski S (2009). Dimethylation of H3K4 by Set1 recruits the Set3 histone deacetylase complex to 5′ transcribed regions.. Cell.

[6] Zhang X, Germann S, Blus BJ, Khorasanizadeh S, Gaudin V (2007). The *Arabidopsis* LHP1 protein colocalizes with histone H3 Lys27 trimethylation.. Nat Struct Mol Biol.

[7] Turck F, Roudier F, Farrona S, Martin-Magniette ML, Guillaume E (2007). Arabidopsis TFL2/LHP1 specifically associates with genes marked by trimethylation of histone H3 lysine 27.. PLoS Genet.

[8] Wang GG, Song J, Wang Z, Dormann HL, Casadio F (2009). Haematopoietic malignancies caused by dysregulation of a chromatin-binding PHD finger.. Nature.

[9] Liu C, Lu F, Cui X, Cao X (2010). Histone methylation in higher plants.. Annu Rev Plant Biol.

[10] Zhao Z, Yu Y, Meyer D, Wu C, Shen WH (2005). Prevention of early flowering by expression of *FLOWERING LOCUS C* requires methylation of histone H3 K36.. Nat Cell Biol.

[11] Cao Y, Dai Y, Cui S, Ma L (2008). Histone H2B monoubiquitination in the chromatin of *FLOWERING LOCUS C* regulates flowering time in *Arabidopsis*.. Plant Cell.

[12] Bastow R, Mylne JS, Lister C, Lippman Z, Martienssen RA (2004). Vernalization requires epigenetic silencing of *FLC* by histone methylation.. Nature.

[13] Jiang D, Wang Y, He Y (2008). Repression of *FLOWERING LOCUS C* and *FLOWERING LOCUS T* by the *Arabidopsis* Polycomb repressive complex 2 components.. PLoS ONE.

[14] Pasini D, Bracken AP, Jensen MR, Lazzerini Denchi E, Helin K (2004). Suz12 is essential for mouse development and for EZH2 histone methyltransferase activity.. EMBO J.

[15] Ringrose L, Paro R (2004). Epigenetic regulation of cellular memory by the Polycomb and Trithorax group proteins.. Annu Rev Genet.

[16] Klose RJ, Kallin EM, Zhang Y (2006). JmjC-domain-containing proteins and histone demethylation.. Nat Rev Genet.

[17] Klose RJ, Yamane K, Bae Y, Zhang D, Erdjument-Bromage H (2006). The transcriptional repressor JHDM3A demethylates trimethyl histone H3 lysine 9 and lysine 36.. Nature.

[18] Shi Y, Lan F, Matson C, Mulligan P, Whetstine JR (2004). Histone demethylation mediated by the nuclear amine oxidase homolog LSD1.. Cell.

[19] Agger K, Cloos PA, Christensen J, Pasini D, Rose S (2007). UTX and JMJD3 are histone H3K27 demethylases involved in *HOX* gene regulation and development.. Nature.

[20] Cloos PA, Christensen J, Agger K, Maiolica A, Rappsilber J (2006). The putative oncogene GASC1 demethylates tri- and dimethylated lysine 9 on histone H3.. Nature.

[21] Whetstine JR, Nottke A, Lan F, Huarte M, Smolikov S (2006). Reversal of histone lysine trimethylation by the JMJD2 family of histone demethylases.. Cell.

[22] Yamane K, Toumazou C, Tsukada Y, Erdjument-Bromage H, Tempst P (2006). JHDM2A, a JmjC-containing H3K9 demethylase, facilitates transcription activation by androgen receptor.. Cell.

[23] Okada Y, Scott G, Ray MK, Mishina Y, Zhang Y (2007). Histone demethylase JHDM2A is critical for Tnp1 and Prm1 transcription and spermatogenesis.. Nature.

[24] He Y, Michaels SD, Amasino RM (2003). Regulation of flowering time by histone acetylation in *Arabidopsis*.. Science.

[25] Jiang D, Yang W, He Y, Amasino RM (2007). *Arabidopsis* relatives of the human lysine-specific Demethylase1 repress the expression of *FWA* and *FLOWERING LOCUS C* and thus promote the floral transition.. Plant Cell.

[26] Liu F, Quesada V, Crevillen P, Baurle I, Swiezewski S (2007). The *Arabidopsis* RNA-binding protein FCA requires a lysine-specific demethylase 1 homolog to downregulate *FLC*.. Mol Cell.

[27] Lu F, Li G, Cui X, Liu C, Wang XJ (2008). Comparative analysis of JmjC domain-containing proteins reveals the potential histone demethylases in *Arabidopsis* and rice.. J Integr Plant Biol.

[28] Jeong JH, Song HR, Ko JH, Jeong YM, Kwon YE (2009). Repression of *FLOWERING LOCUS T* chromatin by functionally redundant histone H3 lysine 4 demethylases in *Arabidopsis*.. PLoS ONE.

[29] Lu F, Cui X, Zhang S, Liu C, Cao X (2010). JMJ14 is an H3K4 demethylase regulating flowering time in *Arabidopsis*.. Cell Res.

[30] Miura A, Nakamura M, Inagaki S, Kobayashi A, Saze H (2009). An *Arabidopsis* jmjC domain protein protects transcribed genes from DNA methylation at CHG sites.. EMBO J.

[31] Noh B, Lee SH, Kim HJ, Yi G, Shin EA (2004). Divergent roles of a pair of homologous jumonji/zinc-finger-class transcription factor proteins in the regulation of *Arabidopsis* flowering time.. Plant Cell.

[32] Saze H, Shiraishi A, Miura A, Kakutani T (2008). Control of genic DNA methylation by a jmjC domain-containing protein in *Arabidopsis thaliana*.. Science.

[33] Searle IR, Pontes O, Melnyk CW, Smith LM, Baulcombe DC (2010). JMJ14, a JmjC domain protein, is required for RNA silencing and cell-to-cell movement of an RNA silencing signal in *Arabidopsis*.. Genes Dev.

[34] Yu X, Li L, Guo M, Chory J, Yin Y (2008). Modulation of brassinosteroid-regulated gene expression by Jumonji domain-containing proteins ELF6 and REF6 in *Arabidopsis*.. Proc Natl Acad Sci U S A.

[35] Li W, Liu H, Cheng ZJ, Su YH, Han HN (2011). DNA Methylation and Histone Modifications Regulate De Novo Shoot Regeneration in *Arabidopsis* by Modulating *WUSCHEL* Expression and Auxin Signaling.. PLoS Genet.

[36] Lu SX, Knowles SM, Webb CJ, Celaya RB, Cha C (2010). The JmjC domain-containing protein JMJ30 regulates period length in the *Arabidopsis* circadian clock.. Plant Physiol.

[37] Baurle I, Dean C (2006). The timing of developmental transitions in plants.. Cell.

[38] Wang JW, Czech B, Weigel D (2009). miR156-regulated SPL transcription factors define an endogenous flowering pathway in *Arabidopsis thaliana*.. Cell.

[39] Wu G, Park MY, Conway SR, Wang JW, Weigel D (2009). The sequential action of miR156 and miR172 regulates developmental timing in *Arabidopsis*.. Cell.

[40] Hayama R, Coupland G (2003). Shedding light on the circadian clock and the photoperiodic control of flowering.. Curr Opin Plant Biol.

[41] Ausin I, Alonso-Blanco C, Jarillo JA, Ruiz-Garcia L, Martinez-Zapater JM (2004). Regulation of flowering time by FVE, a retinoblastoma-associated protein.. Nat Genet.

[42] Baurle I, Dean C (2008). Differential interactions of the autonomous pathway RRM proteins and chromatin regulators in the silencing of *Arabidopsis* targets.. PLoS ONE.

[43] He Y, Doyle MR, Amasino RM (2004). PAF1-complex-mediated histone methylation of *FLOWERING LOCUS C* chromatin is required for the vernalization-responsive, winter-annual habit in *Arabidopsis*.. Genes Dev.

[44] Michaels SD, Amasino RM (1999). *FLOWERING LOCUS C* encodes a novel MADS domain protein that acts as a repressor of flowering.. Plant Cell.

[45] Schmitz RJ, Tamada Y, Doyle MR, Zhang X, Amasino RM (2009). Histone H2B deubiquitination is required for transcriptional activation of *FLOWERING LOCUS C* and for proper control of flowering in *Arabidopsis*.. Plant Physiol.

[46] Johanson U, West J, Lister C, Michaels S, Amasino R (2000). Molecular analysis of *FRIGIDA*, a major determinant of natural variation in *Arabidopsis* flowering time.. Science.

[47] Lempe J, Balasubramanian S, Sureshkumar S, Singh A, Schmid M (2005). Diversity of flowering responses in wild A*rabidopsis thaliana* strains.. PLoS Genet.

[48] Lim MH, Kim J, Kim YS, Chung KS, Seo YH (2004). A new *Arabidopsis* gene, *FLK*, encodes an RNA binding protein with K homology motifs and regulates flowering time via *FLOWERING LOCUS C*.. Plant Cell.

[49] Simpson GG, Dijkwel PP, Quesada V, Henderson I, Dean C (2003). FY is an RNA 3′ end-processing factor that interacts with FCA to control the *Arabidopsis* floral transition.. Cell.

[50] Hornyik C, Terzi LC, Simpson GG (2010). The spen family protein FPA controls alternative cleavage and polyadenylation of RNA.. Dev Cell.

[51] Liu F, Marquardt S, Lister C, Swiezewski S, Dean C (2010). Targeted 3′ processing of antisense transcripts triggers *Arabidopsis FLC* chromatin silencing.. Science.

[52] Swiezewski S, Liu F, Magusin A, Dean C (2009). Cold-induced silencing by long antisense transcripts of an *Arabidopsis* Polycomb target.. Nature.

[53] Sung S, Amasino RM (2004). Vernalization in *Arabidopsis thaliana* is mediated by the PHD finger protein VIN3.. Nature.

[54] Sheldon CC, Conn AB, Dennis ES, Peacock WJ (2002). Different regulatory regions are required for the vernalization-induced repression of *FLOWERING LOCUS C* and for the epigenetic maintenance of repression.. Plant Cell.

[55] Searle I, He Y, Turck F, Vincent C, Fornara F (2006). The transcription factor FLC confers a flowering response to vernalization by repressing meristem competence and systemic signaling in *Arabidopsis*.. Genes Dev.

[56] Jiang D, Gu X, He Y (2009). Establishment of the winter-annual growth habit via FRIGIDA-mediated histone methylation at F*LOWERING LOCUS C* in *Arabidopsis*.. Plant Cell.

[57] Pien S, Fleury D, Mylne JS, Crevillen P, Inze D (2008). ARABIDOPSIS TRITHORAX1 dynamically regulates *FLOWERING LOCUS C* activation via histone 3 lysine 4 trimethylation.. Plant Cell.

[58] Tamada Y, Yun JY, Woo SC, Amasino RM (2009). ARABIDOPSIS TRITHORAX-RELATED7 is required for methylation of lysine 4 of histone H3 and for transcriptional activation of *FLOWERING LOCUS C*.. Plant Cell.

[59] Sung S, He Y, Eshoo TW, Tamada Y, Johnson L (2006). Epigenetic maintenance of the vernalized state in *Arabidopsis thaliana* requires LIKE HETEROCHROMATIN PROTEIN 1.. Nat Genet.

[60] Kardailsky I, Shukla VK, Ahn JH, Dagenais N, Christensen SK (1999). Activation tagging of the floral inducer *FT*.. Science.

[61] Kobayashi Y, Kaya H, Goto K, Iwabuchi M, Araki T (1999). A pair of related genes with antagonistic roles in mediating flowering signals.. Science.

[62] Takada S, Goto K (2003). Terminal flower2, an Arabidopsis homolog of heterochromatin protein1, counteracts the activation of *flowering locus T* by constans in the vascular tissues of leaves to regulate flowering time.. Plant Cell.

[63] He Y, Amasino RM (2005). Role of chromatin modification in flowering-time control.. Trends Plant Sci.

[64] Abe M, Kobayashi Y, Yamamoto S, Daimon Y, Yamaguchi A (2005). FD, a bZIP protein mediating signals from the floral pathway integrator FT at the shoot apex.. Science.

[65] Wigge PA, Kim MC, Jaeger KE, Busch W, Schmid M (2005). Integration of spatial and temporal information during floral induction in *Arabidopsis*.. Science.

[66] Corbesier L, Vincent C, Jang S, Fornara F, Fan Q (2007). FT protein movement contributes to long-distance signaling in floral induction of *Arabidopsis*.. Science.

[67] Jaeger KE, Wigge PA (2007). FT protein acts as a long-range signal in *Arabidopsis*.. Curr Biol.

[68] Mathieu J, Warthmann N, Kuttner F, Schmid M (2007). Export of FT protein from phloem companion cells is sufficient for floral induction in *Arabidopsis*.. Curr Biol.

[69] Yang W, Jiang D, Jiang J, He Y (2010). A plant-specific histone H3 lysine 4 demethylase represses the floral transition in *Arabidopsis*.. Plant J.

[70] Lu F, Cui X, Zhang S, Jenuwein T, Cao X (2011). *Arabidopsis* REF6 is a histone H3 lysine 27 demethylase.. Nat Genet.

[71] Christensen J, Agger K, Cloos PA, Pasini D, Rose S (2007). RBP2 belongs to a family of demethylases, specific for tri-and dimethylated lysine 4 on histone 3.. Cell.

[72] Iwase S, Lan F, Bayliss P, de la Torre-Ubieta L, Huarte M (2007). The X-linked mental retardation gene *SMCX/JARID1C* defines a family of histone H3 lysine 4 demethylases.. Cell.

[73] Li F, Huarte M, Zaratiegui M, Vaughn MW, Shi Y (2008). Lid2 is required for coordinating H3K4 and H3K9 methylation of heterochromatin and euchromatin.. Cell.

[74] Alonso JM, Stepanova AN, Leisse TJ, Kim CJ, Chen H (2003). Genome-wide insertional mutagenesis of *Arabidopsis thaliana*.. Science.

[75] Bonke M, Thitamadee S, Mahonen AP, Hauser MT, Helariutta Y (2003). APL regulates vascular tissue identity in *Arabidopsis*.. Nature.

[76] Imlau A, Truernit E, Sauer N (1999). Cell-to-cell and long-distance trafficking of the green fluorescent protein in the phloem and symplastic unloading of the protein into sink tissues.. Plant Cell.

[77] Clarke JH, Dean C (1994). Mapping *FRI*, a locus controlling flowering time and vernalization response in *Arabidopsis thaliana*.. Mol Gen Genet.

[78] Fletcher JC, Brand U, Running MP, Simon R, Meyerowitz EM (1999). Signaling of cell fate decisions by CLAVATA3 in *Arabidopsis* shoot meristems.. Science.

[79] Hong EH, Jeong YM, Ryu JY, Amasino RM, Noh B (2009). Temporal and spatial expression patterns of nine *Arabidopsis* genes encoding Jumonji C-domain proteins.. Mol Cells.

[80] An H, Roussot C, Suarez-Lopez P, Corbesier L, Vincent C (2004). CONSTANS acts in the phloem to regulate a systemic signal that induces photoperiodic flowering of *Arabidopsis*.. Development.

[81] Yant L, Mathieu J, Schmid M (2009). Just say no: floral repressors help *Arabidopsis* bide the time.. Curr Opin Plant Biol.

[82] Turck F, Fornara F, Coupland G (2008). Regulation and identity of florigen: FLOWERING LOCUS T moves center stage.. Annu Rev Plant Biol.

[83] Kobayashi Y, Weigel D (2007). Move on up, it's time for change–mobile signals controlling photoperiod-dependent flowering.. Genes Dev.

[84] Lee I, Amasino RM (1995). Effect of Vernalization, Photoperiod, and Light Quality on the Flowering Phenotype of *Arabidopsis* Plants Containing the *FRIGIDA* Gene.. Plant Physiol.

[85] Clough SJ, Bent AF (1998). Floral dip: a simplified method for Agrobacterium-mediated transformation of *Arabidopsis thaliana*.. Plant J.

[86] Hemerly AS, Ferreira P, de Almeida Engler J, Van Montagu M, Engler G (1993). *cdc2a* expression in *Arabidopsis* is linked with competence for cell division.. Plant Cell.

[87] Jefferson RA, Kavanagh TA, Bevan MW (1987). GUS fusions: beta-glucuronidase as a sensitive and versatile gene fusion marker in higher plants.. EMBO J.

[88] Bowler C, Benvenuto G, Laflamme P, Molino D, Probst AV (2004). Chromatin techniques for plant cells.. Plant J.

[89] Saleh A, Alvarez-Venegas R, Avramova Z (2008). An efficient chromatin immunoprecipitation (ChIP) protocol for studying histone modifications in Arabidopsis plants.. Nat Protoc.

[90] Wesley SV, Helliwell CA, Smith NA, Wang MB, Rouse DT (2001). Construct design for efficient, effective and high-throughput gene silencing in plants.. Plant J.

