# Supplementary figures and images for: A Companion Cell–Dominant and Developmentally Regulated H3K4 Demethylase Controls Flowering Time in Arabidopsis via the Repression of FLC Expression
Source: PLoS Genet. 2012 Apr 19;8(4):e1002664. doi: 10.1371/journal.pgen.1002664 (PMC3334889; doi:10.1371/journal.pgen.1002664)

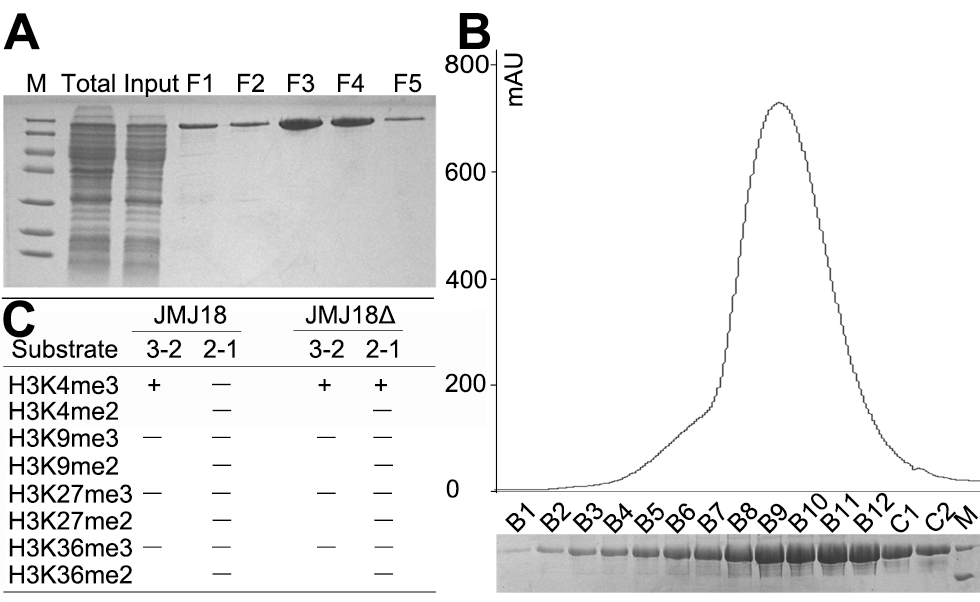

Supplement: Figure S1 — His-JMJ18 purification and characterization of its histone demethylase activity. (A) SDS-PAGE of purified recombinant His-JMJ18 from High5 insect cells. M, molecular standard; Total, total lysate; Input, cleared input. F1–F5, the fractions eluted from the nickel-affinity column. The numbers represent the molecular weights. (B) Purification of His-JMJ18 by size-exclusion chromatography. Top panel, UV absorbance; Bottom panel, results of the SDS-PAGE analysis of the purified and eluted recombinant His-JMJ18 fractions produced by size-exclusion chromatography. (C) Summary of the histone demethylase activity of His-JMJ18 in vitro. (TIF) [file pgen.1002664.s001.tif]

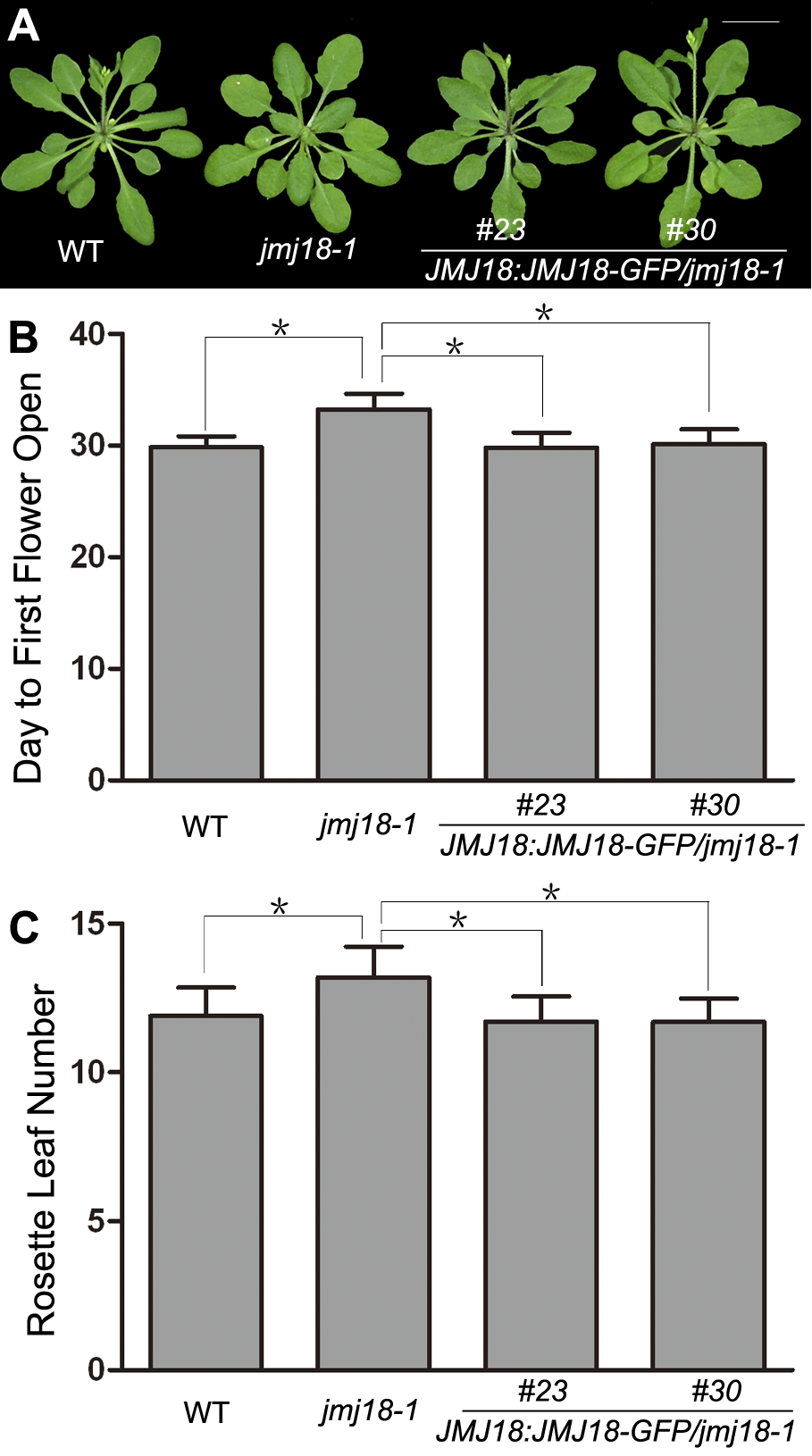

Supplement: Figure S2 — Complementation assay of jmj18-1 by JMJ18:JMJ18-GFP transformation. (A) The late-flowering phenotype of jmj18-1 was complemented by JMJ18:JMJ18-GFP transformation. Twenty-eight-day-old plants were photographed. Bar = 2 cm. (B) and (C) Statistical analysis the phenotype of complementary plants by days to first flower open (B) and rosettle leaf number (C). At least 16 plants of each genotype were used for analysis. Asterisks indicate the significant differences analyzed by Student's t test (P<0.05). (TIF) [file pgen.1002664.s002.tif]

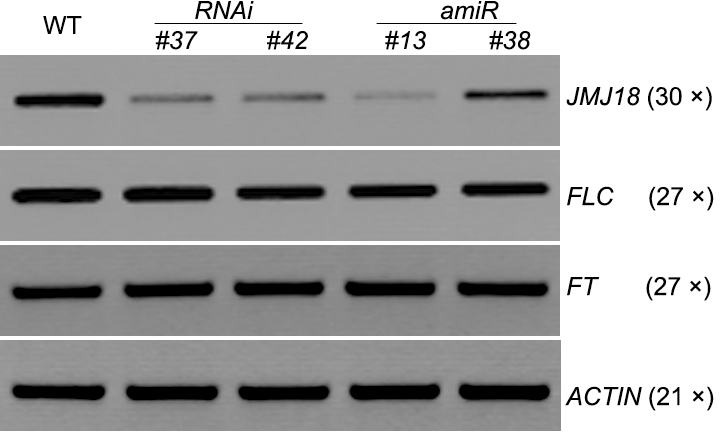

Supplement: Figure S3 — Characterization of JMJ18 RNAi and amiR lines. The gene expression levels of JMJ18, FLC and FT in JMJ18 knock-down transgenic lines. RNAi: knock-down the expression of JMJ18 by double strands of RNA. amiR: knock-down the expression of JMJ18 by artificial microRNA. The numbers in parentheses indicate the amplification cycles. (TIF) [file pgen.1002664.s003.tif]

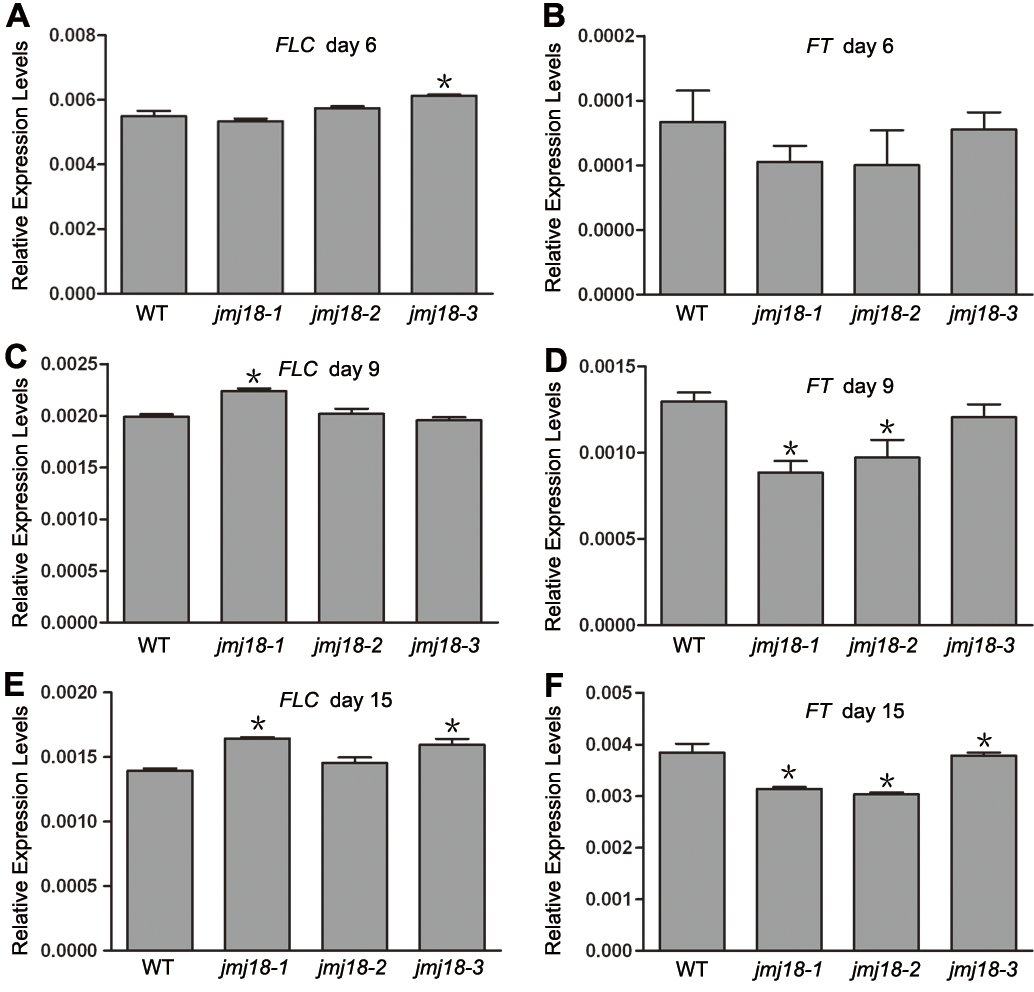

Supplement: Figure S4 — The expression levels of FLC and FT in jmj18 mutants at differential vegetative developmental stages. The expression levels of FLC at day 6 (A), 9 (C) and 15 (E), and FT at day 6 (B), 9 (D) and 15 (F) grown under long-day condition were measured. The eleven-day-old seedlings were collected at dusk at the day indicated. The expression level was normalized to that of ACTIN. Error bars indicate the standard deviation of three replicates. Asterisks indicate the significant difference between wild-type and jmj18 mutants analyzed by Student's t test (P<0.05). (TIF) [file pgen.1002664.s004.tif]

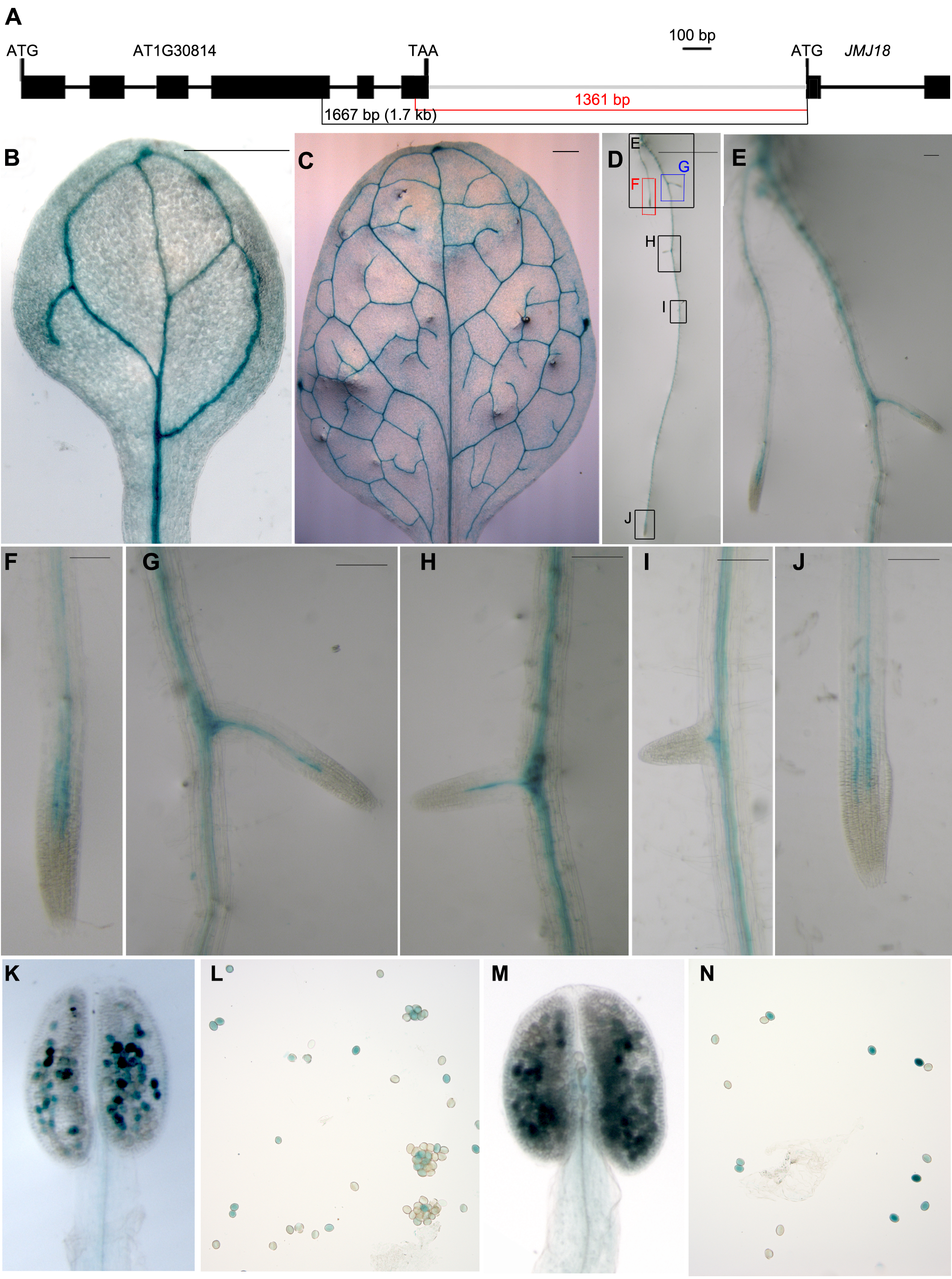

Supplement: Figure S5 — The expression pattern of JMJ18. (A) Two promoter regions selected for GUS constructs. The black filled boxes are shown exons, black lines for introns, and gray lines for intergenic sequence. (B) 7-day-old cotyledon. Bar = 1 mm. (C) 20-day-old rosette leaf. Bar = 1 mm. (D) 10-day-old root. Bar = 0.5 cm. (E)–(J) The marked regions of the root in (D). Bar = 0.1 mm. GUS staining in anther (K, M) and (L, N). 21 independent T2 lines were used for GUS staining. JMJ181.7 kb:GUS transgenic plants were used in (A)–(L). All 21 independent lines we examined display the similar expression pattern for JMJ18. JMJ181.4 kb:GUS transgenic plants were used in (M) and (N). (TIF) [file pgen.1002664.s005.tif]

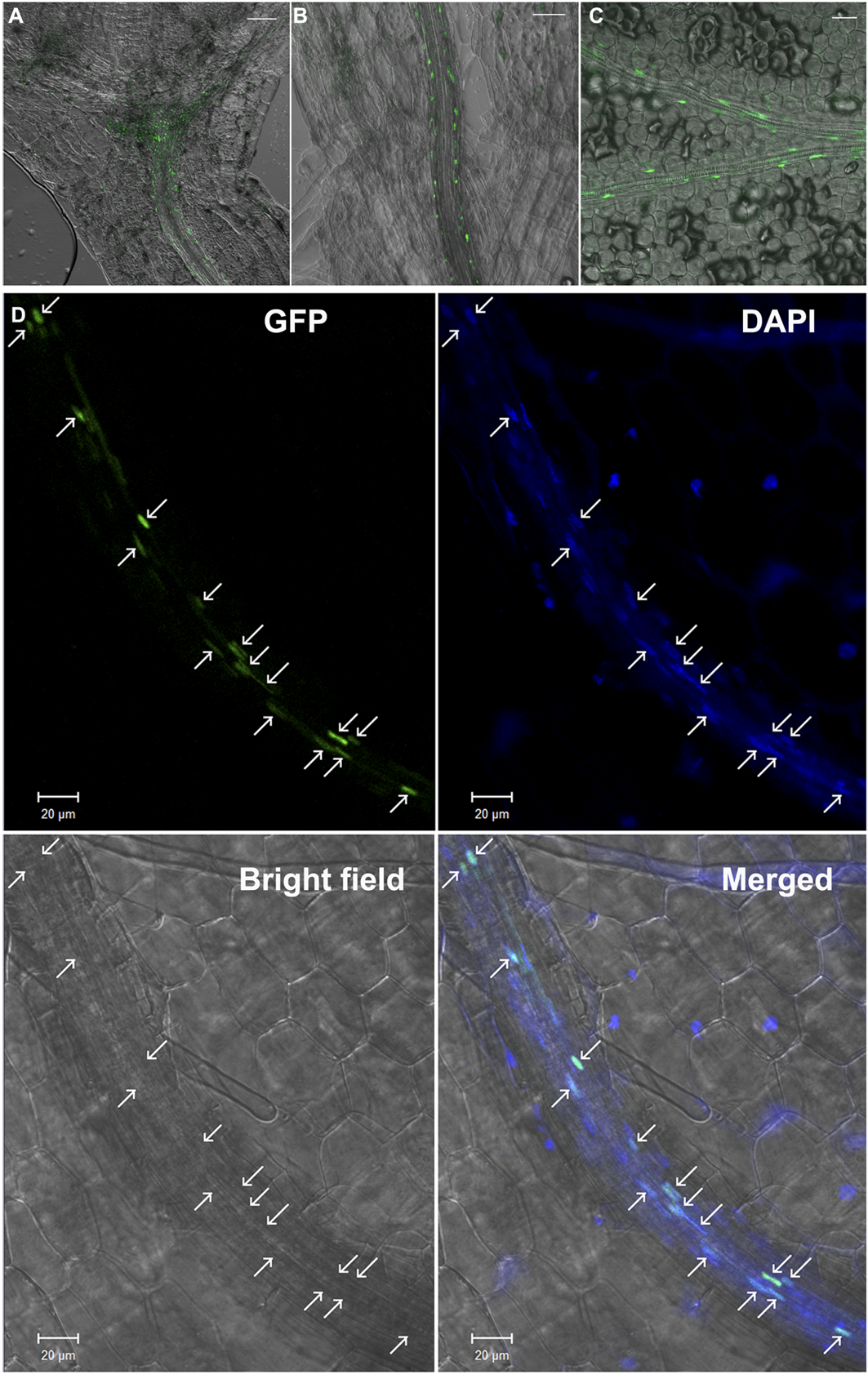

Supplement: Figure S6 — The expression pattern of JMJ18 in stem, hypocotyl and petal by using JMJ181.4 kb:JMJ18-GFP and nuclear localization of JMJ18. JMJ18 was expressed in companion cells in different tissues. GFP fluorescence was detected in (A) the junction region between cotyledon and hypocotyl from 7-day-old seedling, where the vascular tissue branched, bar = 100 µm; (B) 7-day-old hypocotyl, bar = 50 µm and (C) petal, bar = 20 µm, respectively. (D) Nuclear localization of JMJ18. Bar = 20 µm. (TIF) [file pgen.1002664.s006.tif]

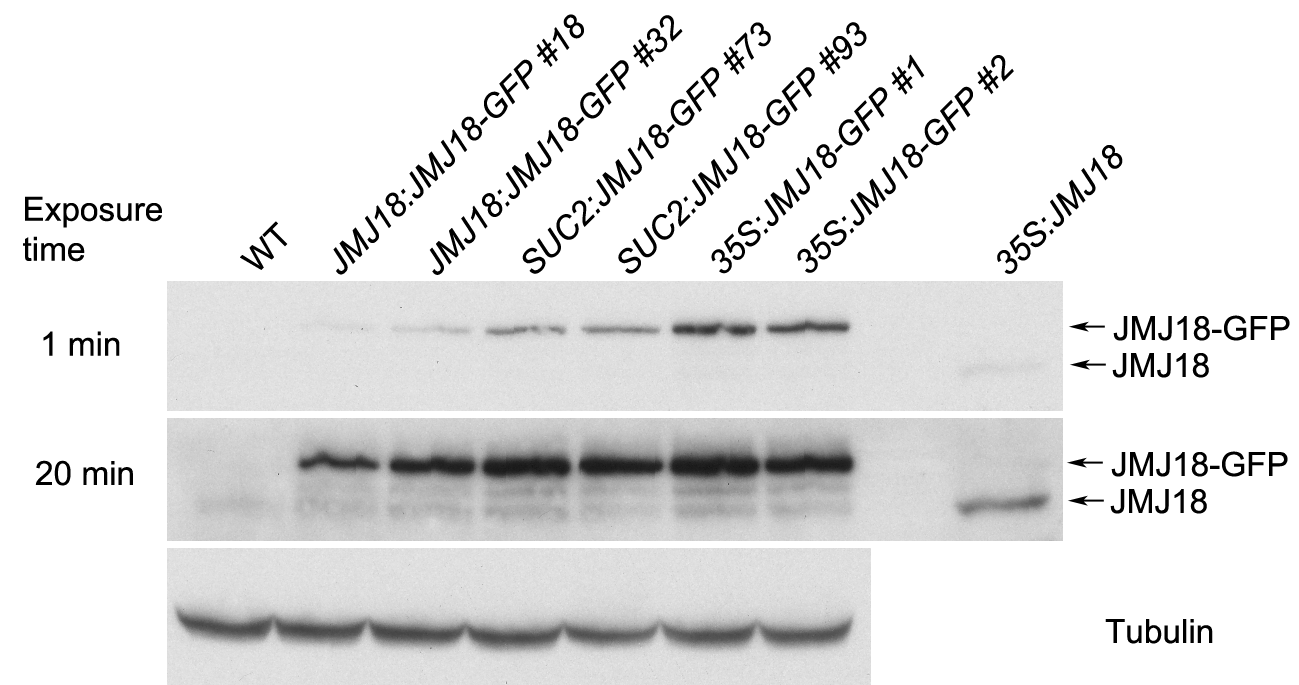

Supplement: Figure S7 — JMJ18 in JMJ18 overexpression and wild-type plants. The 35:JMJ18 transgenic plant was used to mark JMJ18 band, about 20% amount of the other samples was loaded for the sample from 35:JMJ18 transgenic plant. The arrows indicated the JMJ18-GFP or JMJ18 bands. The upper panel: the gel was exposure for 1 min; the lower panel: the gel was exposure for 20 min. Tubulin was used as a loading control. (TIF) [file pgen.1002664.s007.tif]

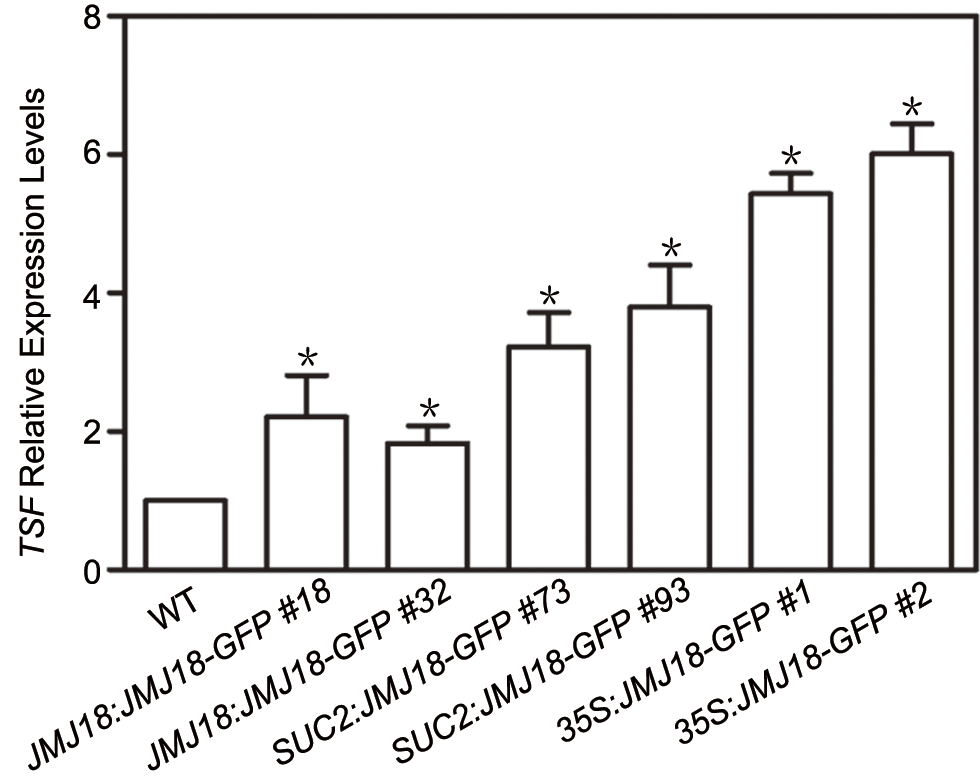

Supplement: Figure S8 — JMJ18 induced TSF expression. qRT-PCR analysis of TSF mRNA expression in the transgenic plants. The expression level was normalized to that of ACTIN. Error bars indicate the standard deviation of three independent biological replicates. Asterisks indicate the significant difference between wild-type and transgenic plants analyzed by Student's t test (P<0.05). (TIF) [file pgen.1002664.s008.tif]

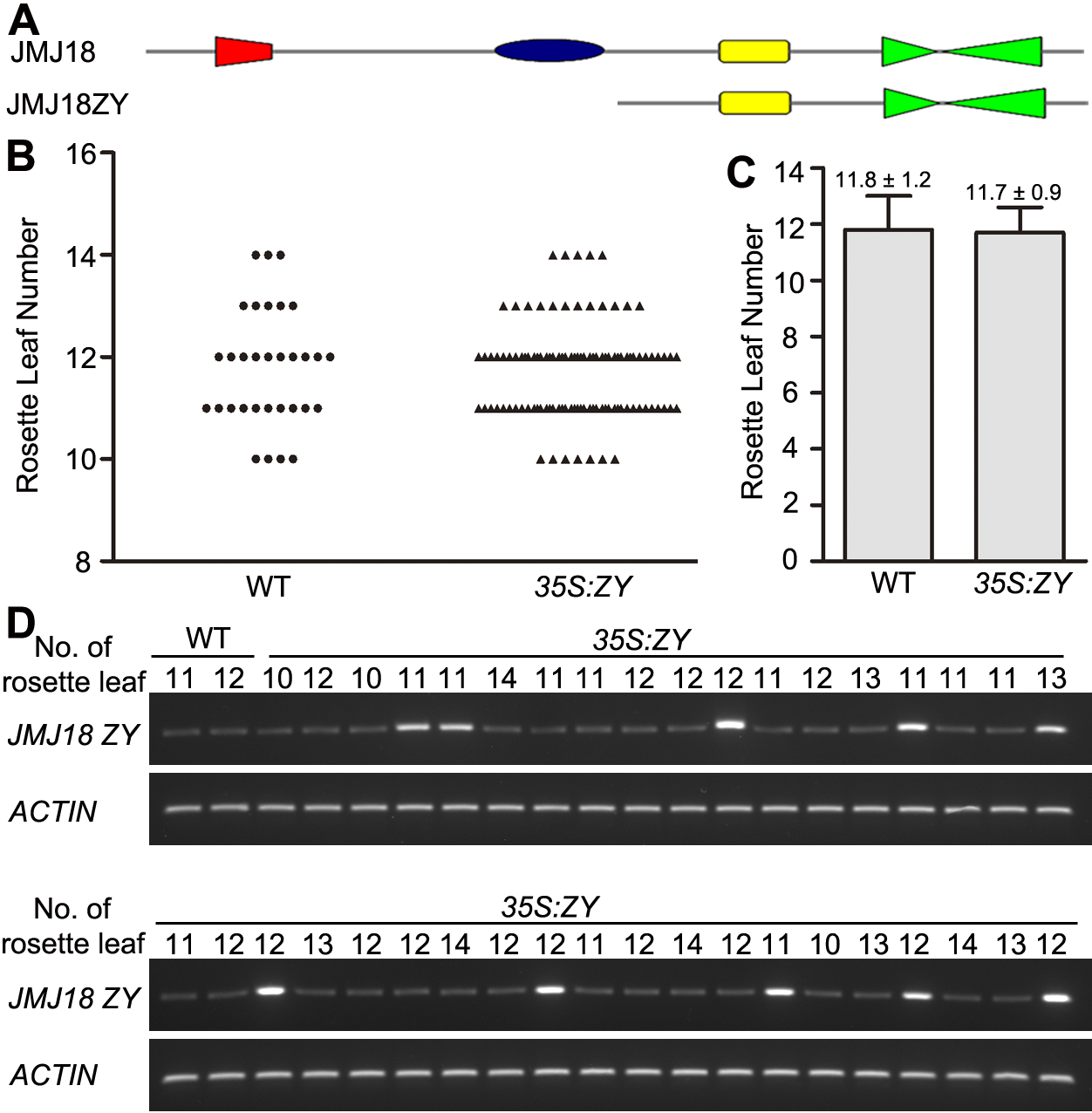

Supplement: Figure S9 — Characterizing 35S:ZY transgenic plants. (A) Schematic structures of full-length JMJ18 protein as well as JMJ18 ZY. (B) Flowering time distributions of wild-type and 35S:ZY at T1 generation measured by rosette leaf number. Thirty-two wild-type and 108 independent transgenic plants were analyzed. (C) Flowering time of wild-type and 35S:ZY transgenic plants counted by rosette leaf number. (D) JMJ18 ZY transcription levels in independent 35S:ZY transgenic line, and the rosette leaf number for each plant was indicated. (TIF) [file pgen.1002664.s009.tif]

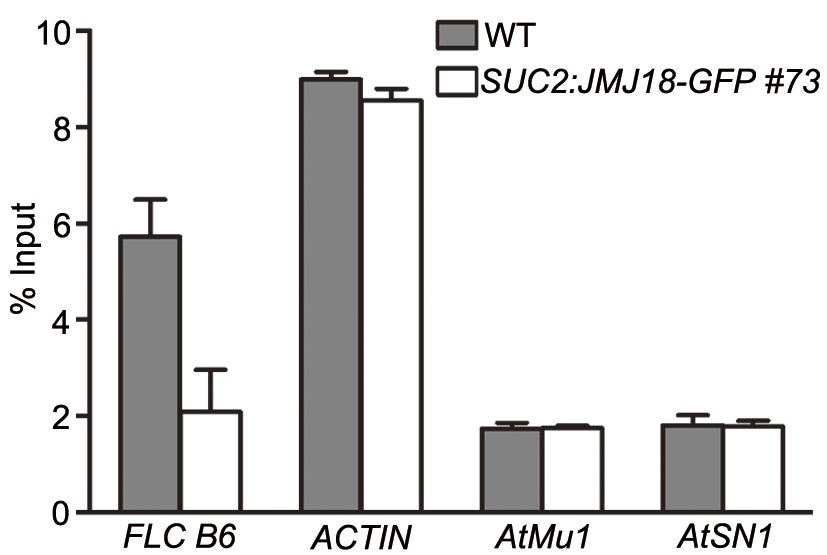

Supplement: Figure S10 — JMJ18 do not affect the H3K4me3 modification levels at ACTIN, AtMu1 and AtSN1 loci. The H3K4me3 modification levels were detected in wild-type and SUC2:JMJ18-GFP plants. The results were normalized to the input. The values are the mean ± standard deviation from three biological replicates. Asterisk indicates the significant difference between wild-type and transgenic plants analyzed by Student's t test (P<0.05). (TIF) [file pgen.1002664.s010.tif]

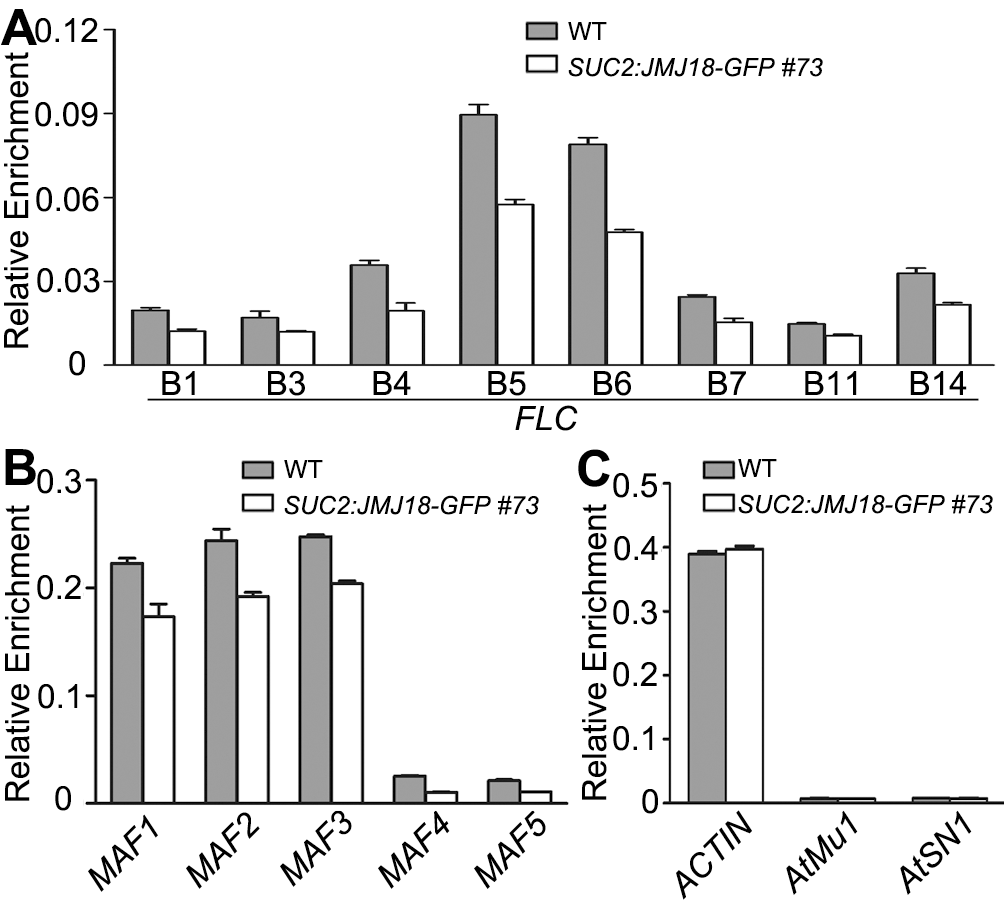

Supplement: Figure S11 — JMJ18 decreases FLC/MAFs H3K4me3 levels. (A) Overexpression JMJ18 reduced H3K4me3 level at FLC locus in JMJ18 overexpression plant compared to wild-type plant. (B) ChIP analysis of H3K4me3 levels for other members of FLC clade genes. (C) The H3K4me3 levels were not obviously changed at ACTIN, AtMu1 or AtSN1 chromatin between wild-type and JMJ18 overexpression plants. The enrichments were normalized to total H3. The values are the mean ± standard deviation from three biological replicates. (TIF) [file pgen.1002664.s011.tif]

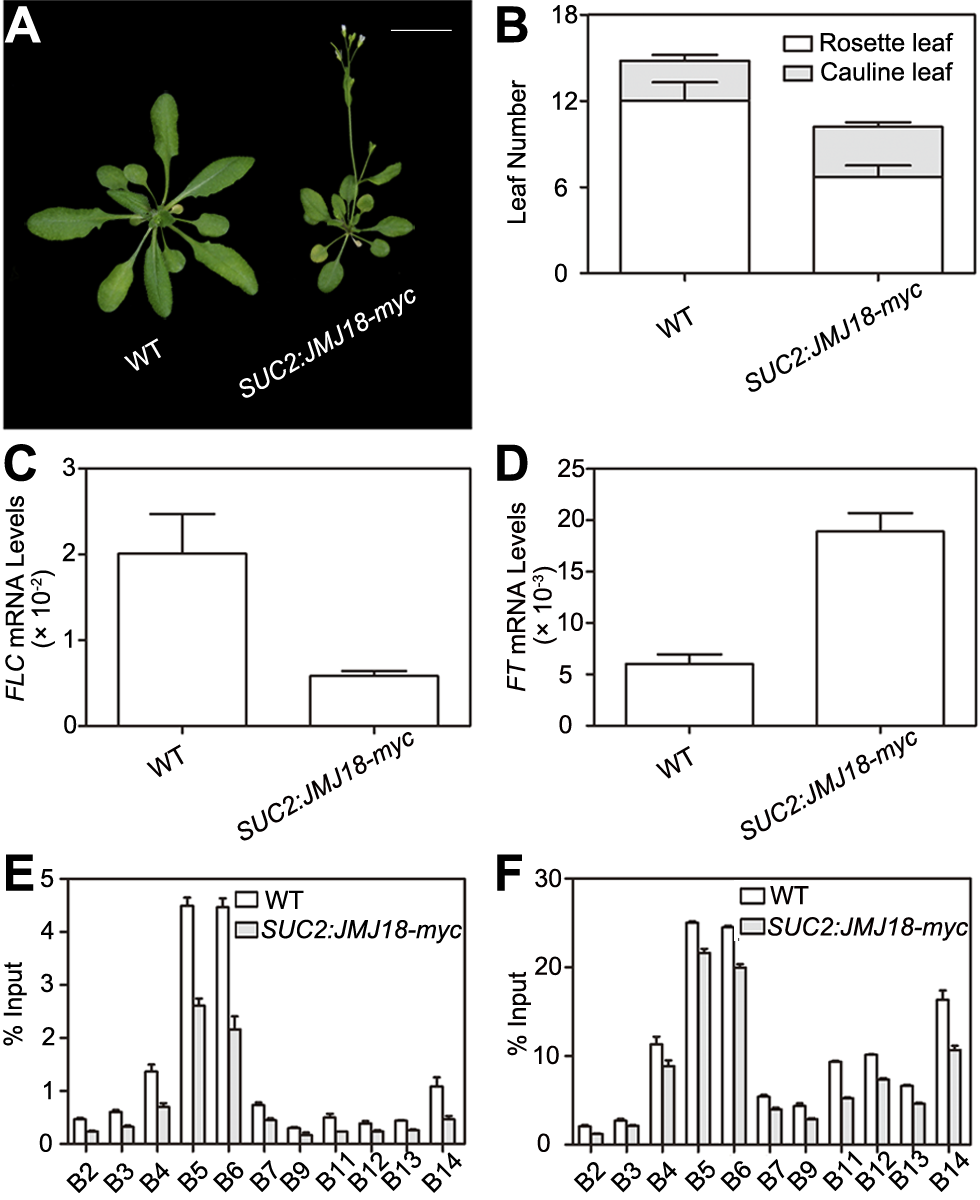

Supplement: Figure S12 — Early-flowering phenotype of the SUC2:JMJ18-myc line. (A) Twenty-four-day-old SUC2:JMJ18-myc plants grown under LD conditions were shown. Bar = 2 cm. Thirty-three out of 108 independent SUC2:JMJ18-myc lines displayed the early-flowering phenotype. The independent line shown was used to analyze FLC and FT expression and for ChIP. (B) Flowering time in wild-type plants and the SUC2:JMJ18-myc line based on the number of rosette leaves at flowering. The values are the mean ± standard deviation from 20 plants. qRT-PCR analysis of FLC (C) and FT (D) expression. The expression level was normalized to that of ACTIN. The levels of H3K4me3 (E) and H3K4me2 (F) across the FLC genomic region in wild-type and SUC2:JMJ18-myc plants were determined. The values in (C) to (F) are the mean and standard deviation from three technical replicates. (TIF) [file pgen.1002664.s012.tif]
