# Supplementary material for: A Companion Cell–Dominant and Developmentally Regulated H3K4 Demethylase Controls Flowering Time in Arabidopsis via the Repression of FLC Expression
Source: PLoS Genet. 2012 Apr 19;8(4):e1002664. doi: 10.1371/journal.pgen.1002664 (PMC3334889; doi:10.1371/journal.pgen.1002664)
Supplement: Table S1 — Knock-down JMJ18 expression leads to late-flowering phenotype. All the plants were grown in LD condition. The values are the mean ± standard deviation. n indicates the plants number scored for the analysis. Asterisks indicate the significant differences in the statistic analysis between wide type and mutants using Student's t test (P<0.05). (DOC) [file pgen.1002664.s013.doc]

**Table S1. Knock-down *JMJ18* expression leads to late-flowering phenotype**

| Genotype | Days to  visible buds | Days to first  flower open | Rosette  leaf no. | Cauline  leaf no. | *n* |
| --- | --- | --- | --- | --- | --- |
| WT | 22.7±1.3 | 29.4±0.7 | 11.7±1.2 | 2.9±0.7 | 35 |
| *RNAi #37* | 24.6±1.2^＊^ | 31.5±1.1^＊^ | 12.9±1.0^＊^ | 3.0±0.7 | 32 |
| *RNAi #42* | 24.9±1.4^＊^ | 31.3±1.0^＊^ | 12.8±0.8^＊^ | 2.8±0.6 | 33 |
| *amiR #13* | 25.5±0.9^＊^ | 31.8±1.3^＊^ | 13.4±0.9^＊^ | 3.1±0.4 | 33 |
| *amiR #38* | 24.0±1.7^＊^ | 30.9±1.7^＊^ | 12.5±1.1^＊^ | 2.9±0.6 | 34 |

All the plants were grown in LD condition. The values are the mean ± standard deviation. *n* indicates the plants number scored for the analysis. Asterisks indicate the significant differences in the statistic analysis between wide type and mutants using Student’s *t* test (P < 0.05).
