# Supplementary material for: A Companion Cell–Dominant and Developmentally Regulated H3K4 Demethylase Controls Flowering Time in Arabidopsis via the Repression of FLC Expression
Source: PLoS Genet. 2012 Apr 19;8(4):e1002664. doi: 10.1371/journal.pgen.1002664 (PMC3334889; doi:10.1371/journal.pgen.1002664)
Supplement: Table S2 — Genetic interaction between JMJ18 and FLC in flowering time control. All the plants were grown under long-day condition. The values are the mean ± standard deviation. n indicates the plant number scored for phenotype analysis. (DOC) [file pgen.1002664.s014.doc]

**Table S2. Genetic interaction between *JMJ18* and *FLC* in flowering time control**

| Genotype | Days to  visible buds | Days to first  flower open | Rosette  leaf no. | Cauline  leaf no. | *n* |
| --- | --- | --- | --- | --- | --- |
| WT | 23.5±0.7 | 30.2±0.9 | 11.4±0.8 | 2.9±0.3 | 36 |
| *jmj18-1* | 25.1±0.7 | 31.8±1.0 | 13.6±0.6 | 3.1±0.3 | 34 |
| *flc-3* | 21.1±0.6 | 27.8±0.8 | 9.7±0.5 | 2.8±0.6 | 35 |
| *jmj18-1 flc-3* | 21.5±0.8 | 28.1±0.6 | 10.1±0.7 | 2.8±0.9 | 35 |
| WT | 23.0±0.8 | 29.9±1.0 | 12.0±0.9 | 2.7±0.5 | 21 |
| *SUC2:JMJ18-GFP #73* | 17.5±0.6 | 22.8±1.1 | 6.9±0.9 | 3.2±0.4 | 21 |
| *flc-3* | 20.5±0.9 | 27.2±1.3 | 10.0±0.8 | 2.6±0.5 | 23 |
| *SUC2:JMJ18-GFP #73* *flc-3* | 17.8±0.5 | 22.6±0.9 | 7.0±0.8 | 3.1±0.7 | 25 |

All the plants were grown under long-day condition. The values are the mean ± standard deviation. *n* indicates the plant number scored for phenotype analysis.
