# Supplementary material for: A Companion Cell–Dominant and Developmentally Regulated H3K4 Demethylase Controls Flowering Time in Arabidopsis via the Repression of FLC Expression
Source: PLoS Genet. 2012 Apr 19;8(4):e1002664. doi: 10.1371/journal.pgen.1002664 (PMC3334889; doi:10.1371/journal.pgen.1002664)
Supplement: Table S3 — The primers used for detecting gene expression level. (DOC) [file pgen.1002664.s015.doc]

**Table S3. The primers used for detecting gene expression level**

| Name | Forward 5’-3’ | Reverse 5’-3’ |
| --- | --- | --- |
| *ACTIN* | GGTGTCATGGTTGGTATGGGTC | CCTCTGTGAGTAGAACTGGGTGC |
| *FT* | TAGTAAGCAGAGTTGTTGGAGACG | GGGAAGGCCGAGATTGTAGAT |
| *FLC* | CCTCTCCGTGACTAGAGCCAAG | AGGTGACATCTCCATCTCAGCTTC |
| *CO* | TAAGGATGCCAAGGAGGTTG | CCCTGAGGAGCCATATTTGA |
| *MAF1* | TCACCTTAAACTCAAAGCCTGATTC | CAAACTCTGATCTTGTCTCCGAAG |
| *MAF2* | CATTGTGGGTCTCCGGTGATTAG | GATGAGACCATTGCGTCGTTTG |
| *MAF3* | TATCTTCCTCGCGCCAATG | AGCACAAGAACTCTGATATTTGTCTAC |
| *MAF4* | GCTTCTCAAGTAACCACCATCAC | CTTGGATGACTTTTCCGTAGCAG |
| *MAF5* | CATGGATTGTGCTAGAAAACAACTG | GCTTCACTCTTCCGACACATCTAATC |
