# Supplementary material for: A Companion Cell–Dominant and Developmentally Regulated H3K4 Demethylase Controls Flowering Time in Arabidopsis via the Repression of FLC Expression
Source: PLoS Genet. 2012 Apr 19;8(4):e1002664. doi: 10.1371/journal.pgen.1002664 (PMC3334889; doi:10.1371/journal.pgen.1002664)
Supplement: Table S4 — The primers used in ChIP assay. (DOC) [file pgen.1002664.s016.doc]

**Table S4. The primers used in ChIP assay**

| Name | Forward 5’-3’ | Reverse 5’-3’ |
| --- | --- | --- |
| *FLC* chipB1 | GCATTAGGTTGTTCCCTCCAAAC | GCCCTACCCATGACTAACGTGAG |
| *FLC* chipB2 | CGGAGTGGGTGAAACTGATTACTG | CATCAAAACTTCTTGGCACAGCTC |
| *FLC* chipB3 | GTTCGGGAGATTAACACAAATAATAAAGG | GAAAACAAGCTGATACAAGCATTTCAC |
| *FLC* chipB4 | TGGGGGTAAACGAGAGTGATG | GCAATAGTTCAATCCGTATCGTAGG |
| *FLC* chipB5 | TGTTCTCAATTCGCTTGATTTCTAGT | GCCCGACGAAGAAAAAGTAGATAG |
| *FLC* chipB6 | CGAGCACGCATCAGATCG | GGCGGATCTCTTGTTGTTTCTC |
| *FLC* chipB7 | GACGTGCATATACAAATCCAAGAGAAC | CTTTGAATCACAATCGTCGTGTG |
| *FLC* chipB8 | GCTGGACCTAACTAGGGGTGAAC | CCTCTTTGGTACGGATCTATAATGAATC |
| *FLC* chipB9 | CTTGAGGACAAGGTTTTTTCCAG | GGCTTCCTCATACTTATGGTTATCTG |
| *FLC* chipB10 | CCTCTACTGTCCAGATTGTTTCTATGC | TGTCATCACATTGTGGCTCATC |
| *FLC* chipB11 | CATCTCTCCAGCCTGGTCAAG | GGGCTATGAAAATTGCGGTATG |
| *FLC* chipB12 | CCTCTCCGTGACTAGAGCCAAG | CTTCAACATGAGTTCGGTCTGC |
| *FLC* chipB13 | CCGGTTGTTGGACATAACTAGG | CTCTACCAAACCCAGACTTAACCAG |
| *FLC* chipB14 | CCTTGGATAGAAGACAAAAAGAGAAAGTG | AGGTGACATCTCCATCTCAGCTTC |
| *MAF1* chip | CTGTATGAAGATAGCAGATGCGTAAG | CAACATCAAGAACGAATCACCAC |
| *MAF2* chip | CTGTATGAGGAGAGCTGATGCTTTAG | GTGAACAACAGATGAATTAGCGATTC |
| *MAF3* chip | TCTTGGTGTTCGTTTCGTGC | TGAACAACAGATGAATTAGCGACAG |
| *MAF4* chip | CAATGTGGTGTCCAAAGTCCAG | GGCTTGAAATCCCAAGACAAA |
| *MAF5* chip | GTGGTGGCGGAAGATGTACTC | CAAGACAAAGTTAAGATGACTTGAAATC |
| *ACTIN* chip | GATCCGTTCGCTTGATTTTGC | ACAAGCACGGATCGAATCACA |
| *AtSN1* chip | ACCAACGTGCTGTTGGCCCAGTGGTAAATC | AAAATAAGTGGTGGTTGTACAAGC |
| *AtMu1* chip | CCGAGAACTGGTTGTGGTTT | GCTCTTGCTTTGGTGATGGT |
